# Quality Control of *Oleum Cinnamomi* Assisted by Network Pharmacology Strategy

**DOI:** 10.3390/molecules27196391

**Published:** 2022-09-27

**Authors:** Lin Zheng, Yang Zhou, Ting Yan, Zipeng Gong, Yueting Li, Siying Chen, Yong Huang, Mingyan Chi

**Affiliations:** 1 State Key Laboratory of Functions and Applications of Medicinal Plants, Guizhou Provincial Key Laboratory of Pharmaceutics, Guizhou Medical University, 4 Beijing Road, Guiyang 550004, China; 2 School of Pharmacy, Guizhou Medical University, 4 Beijing Road, Guiyang 550004, China

**Keywords:** *Oleum Cinnamomi*, essential oil, coronary heart disease, network pharmacology, quality control

## Abstract

*Oleum Cinnamomi* is a traditional medicine used by the Hmong, the essential oil obtained from *Fructus Cinnamomi*, for the treatment of coronary heart disease. Information regarding the efficient quality control markers of it is lacking, which has become a bottleneck restricting its development and utilization. Here, an integrated qualitative analysis approach based on a GC-MS and network pharmacology strategy was applied to explore quality control markers for the assessment of *Oleum Cinnamomi*. Firstly, the compounds of *Oleum Cinnamomi* were detected by GC-MS. In total, 57 chemical components were identified, mainly monoterpenes and sesquiterpenes, accounting for 83.05% of total essential oil components. Secondly, network pharmacology was adopted to explore the compounds linked to target genes of coronary heart disease. Fifty-two compounds were found, indicating the effectiveness of *Oleum Cinnamomi* in the treatment of coronary heart disease. Among them, 10 compounds, including eucalyptol, were chosen as potential effective compounds in *Oleum Cinnamomi*. Thirdly, an established GC-MS SIM method was validated and applied for the simultaneous determination of the contents of these 10 compounds using 20 sample batches of *Oleum Cinnamomi*. It was preliminarily found that the contents of these 10 compounds differed in *Oleum Cinnamomi* from different origins. Finally, quantitative analyte data were analyzed using multivariate statistical analysis to determine *Oleum Cinnamomi* quality. Four compounds (eucalyptol, *p*-cymene, sabinene, β-pinene) were identified as chemical markers for quality control. Accordingly, this study provides new strategies to explore the quality control markers and develops a novel method for the quality assessment of *Oleum Cinnamomi*.

## 1. Introduction

*Oleum Cinnamomi* is the essential oil obtained from *Fructus Cinnamomi* by steam distillation and is included in the “Quality standard of traditional Chinese medicinal materials and ethnic medicinal materials in Guizhou Province” [1]. In addition, it was first recorded in the “Supplement to Compendium of Materia Medica” [2]. *Fructus Cinnamomi* is the dry and ripe fruit of *Cinnamomum migao* H.W.Li, also known as Maodanmu, Qingtumu, and octagonal belt. It is produced in Guizhou, Guangxi, and Yunnan. *Fructus Cinnamomi* is widely used to treat heart disease, stomach pain, abdominal pain, chest pain, chest tightness, and other ailments, and the essential oil extracted from this medicine is the main pharmacodynamic component [3]. Modern pharmacological studies have shown that *Oleum Cinnamomi* can increase myocardial oxygen supply, reduce myocardial oxygen consumption, exert anti-myocardial ischemia effects, and relax visceral smooth muscle [4,5,6]; moreover, it also has anti-viral [7], anti-inflammatory and analgesic [8], antibacterial [9], and other effects.

The therapeutic mechanism of traditional Chinese medicine (TCM) with regard to linked disorders can be fully and systematically described using the network pharmacology approach, which can be utilized to characterize “multiple compounds, multiple targets, and multiple pathways” [10,11]. Network pharmaceutical analysis has been widely used to anticipate the probable active ingredients in herbs and research the potential therapeutic mechanisms of TCMs, such as the GuanXinNing tablet, Huanglian Jiedu decoction, and Toujie Quwen granule [12,13,14]. Although network pharmacological analysis had previously been applied to *Oleum Cinnamomi*, the number of compounds selected was too small to predict the correct signaling pathway [6]. Several previous studies have found that compounds such as eucalyptol, β-pinene and limonene were the main compounds of *Oleum Cinnamomi* by GC-MS analysis, but there was insufficient evidence to list them as quality markers [15,16,17]. Therefore, it is imperative to find the compounds capable of quality control in *Oleum Cinnamomi*.

At present, quality control for TCM is more than adequate for safety but insufficient in terms of efficacy. Therefore, the pharmacological activity of a component should also be considered when determining the quality control index [18]. In this study, an integrated qualitative analysis approach based on a GC-MS and network pharmacology strategy was firstly applied to explore quality control markers of *Oleum Cinnamomi*. Through matching the measured fragment information of *Oleum Cinnamomi* from GC-MS with the NIST 2020 standard mass spectrometry library, a total of 57 main compounds were identified. Next, according to the clinical efficacy of *Oleum Cinnamomi* and the identified compounds, a network pharmacology method was used to discover the potential active compounds and a "chemical–gene–pathway” network was constructed. Subsequently, a novel method for simultaneously detecting the 10 potential active compounds was developed by GC-MS SIM and applied to assess the quality of 20 batches of *Oleum Cinnamomi*. It was preliminarily found that the contents of these 10 compounds were different in *Oleum Cinnamomi* from different origins. Finally, quantitative analyte data were analyzed using multivariate statistical analysis to determine *Oleum Cinnamomi* quality. Four compounds of eucalyptol, *p*-cymene, sabinene, β-pinene were selected as chemical markers for quality control. Accordingly, this study provides new strategies to explore the quality control markers and develops a novel method for the quality assessment of *Oleum Cinnamomi*.

## 2. Results and Discussion

### 2.1. Optimal Conditions for Sample Pretreatment and the Apparatus

To improve the oil yield of herbs, single-factor investigation was carried out based on the influence of the crushing particle size (10, 30, 60, 80, 100 mesh), solid–liquid ratio (1:6, 1:8, 1:10, 1:12, 1:14 g/mL), soaking time (0, 0.5, 1, 1.5, 2 h), and extraction time (2, 3, 4, 5, 6 h). It is theoretically believed that a smaller particle size and larger surface area are beneficial for penetration of the solvent, as the diffusion distance is shorter, which is optimal for diffusion of the components. However, for the particle size investigated, the oil yield of medicinal herbs was highest when the crushing particle size was 60 mesh rather than 100 mesh. This could be because the oversized surface area increases its adsorption capacity, and when the pulverized components are too fine, many macromolecular peptizing substances in the cells dissolve out. This phenomenon increases the surface viscosity of the medicinal powder and reduces the diffusion coefficient, thereby reducing the oil yield. Further, when the solid–liquid ratio was 1:8 (g/mL), the soaking time was 0.5 h and the extraction time was 5 h, and the oil yield of the medicinal material was highest after screening. For the specific parameters of each part, see the Supplementary Material “Screening of Extraction Methods” section in the Supplementary Materials.

Compared to the GC-MS SCAN mode, the GC-MS SIM mode allows the mass spectrometer to spend more time detecting the ion current of selected mass-to-charge ratio ions, thereby increasing the analytical sensitivity. This method is widely used in research fields such as medicine, food, and environmental monitoring [19]. Therefore, the target ions and reference ions of the index components of *Oleum Cinnamomi* were selected to establish the GC-MS SIM analysis conditions using GC-MS SCAN mode. In addition, taking the baseline stability, resolution, and chromatographic peak shape as evaluation indicators, the SH-Stabilwax (30 m × 0.32 mm, 0.25 μm film thickness, Shimadzu Co., Ltd., Kyoto, Japan), SH-Rxi-17Sil MS (30 m × 0.25 mm, 0.25 μm film thickness, Shimadzu Co., Ltd., Kyoto, Japan), SH-Rxi-5Sil MS (30 m × 0.25 mm, 0.25 μm film thickness, Shimadzu Co., Ltd., Kyoto, Japan), and other chromatographic columns were mainly investigated in this experiment for the separation of the chemical constituents of *Oleum Cinnamomi*. Meanwhile, solvents (methanol, ethanol, ethyl acetate, *n*-hexane, cyclohexane) and internal standards (cyclohexanone, naphthalene, benzene, xylene, 2-butanol, dioctyl phthalate, dimethyl phthalate, diethyl phthalate, and dibutyl phthalate) were investigated. The results showed that the best experimental conditions were as follows: column, SH-Stabilwax (30 m × 0.32 mm, 0.25 μm film thickness); solvent, ethyl acetate; and internal standard, a mixture of cyclohexanone and naphthalene. Finally, the contents of 10 active components were determined based on this condition.

### 2.2. GC-MS to Identify Components of Oleum Cinnamomi

**Figure 1:**
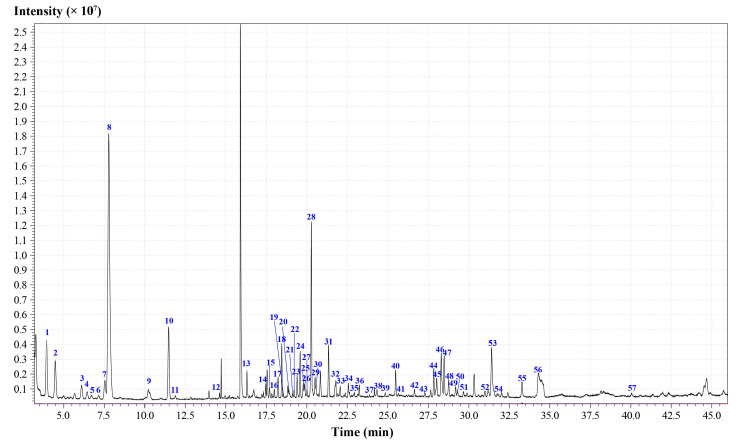
GC-MS total ion chromatograph of *Oleum Cinnamomi*. Note: The compounds represented by the numbers were consistent with those in Table 1.

**Figure 2:**
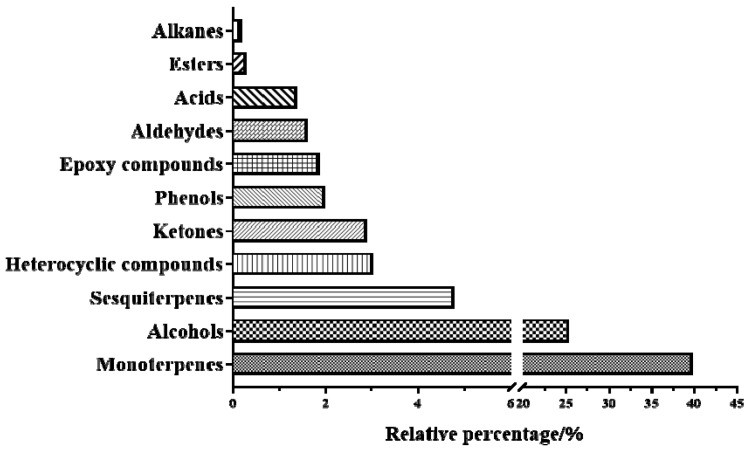
Relative percent contents of various compounds in *Oleum Cinnamomi*.

The test products were prepared according to the method described in Section 3.3 for the identification of chemical components in *Oleum Cinnamomi* (Batch: S9). The injection volume was 1.0 μL. The GC-MS total ion chromatograph (Figure 1) was obtained according to the sampling analysis based on the apparatus conditions (Section 3.4). The measured fragment information was matched with the NIST 2020 standard mass spectrometry library, and the peaks with a matching degree greater than 80 were selected. Meanwhile, the relevant literature was combined to increase the reliability of the identification results. Based on these, 57 chemical constituents were identified in the essential oil of this herb. The relative percent contents of the 57 components (Table 1) in the essential oil were calculated by the peak area normalization method, and the relative percentage of this was found to be 83.05%. The relative percentages of the various compounds are shown in Figure 2, including 11 monoterpenes (39.80%), 21 alcohols (25.31%), 4 sesquiterpenes (4.77%), 6 heterocyclic compounds (3.02%), 4 ketones (2.89%), 1 phenol (1.97%), 2 epoxy compounds (1.85%), 3 aldehydes (1.6%), 3 acids (1.38%), 1 ester (0.28%), and 1 alkane (0.18%). The chemical components with relatively high contents in the essential oil of this herb were as follows: eucalyptol (25.03%), α-terpineol (6.23%), *p*-cymene (4.66%), agarospirol (3.59%), *β*-pinene (3.53%), β-cadinene (2.6%), sabinene (2.53%), and rosifoliol (2.48%), among others. In addition, in order to verify the accuracy of qualitative analysis results, as many reference substances as possible were obtained to confirm the identification results. As a result, a total of 21 components were confirmed by comparison with the standard compounds (Supplementary Figure S1).

### 2.3. Network Pharmacological Analysis of Oleum Cinnamomi

**Figure 3:**
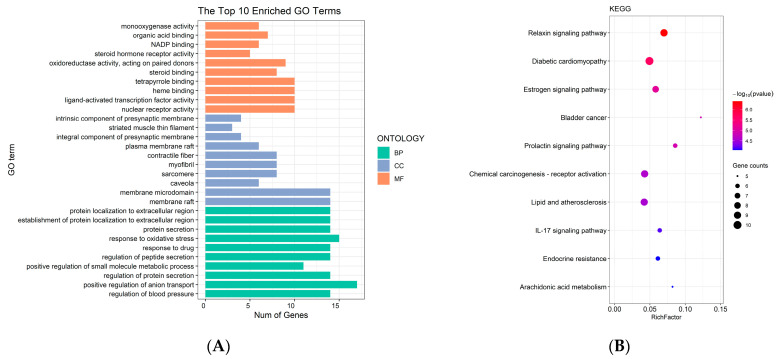
Intersecting targets of *Oleum Cinnamomi* with respect to the treatment of coronary heart disease for GO analysis (**A**) (BP: biological process; CC: cellular component; MF: molecular function); and KEGG enrichment analysis (**B**).

**Figure 4:**
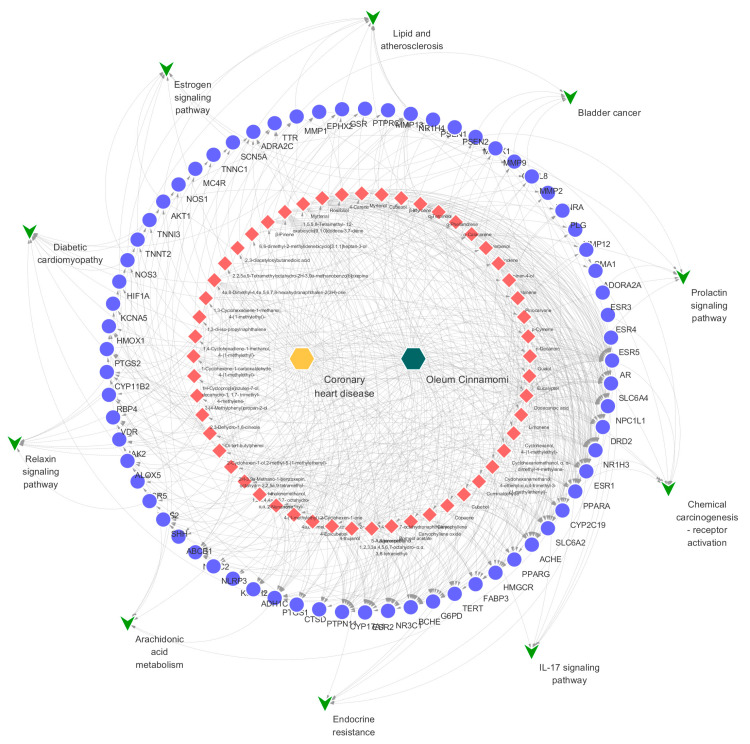
Chemical–target–pathway network of *Oleum Cinnamomi* (green arrows represent pathways; blue circles represent target genes; red diamonds represent chemicals; and orange and dark green hexagons represent drugs and diseases, respectively).

The chemical-target gene database was created by compiling 258 target genes that were discovered to interact with the 57 identified compounds (Supplementary Table S1). The disease-target gene database was created with 1040 genes after the target genes of coronary heart disease were searched in the Therapeutic Target Database, OMIM, DisGeNet, and GeneCards (Supplementary Table S2). Finally, by comparing the two aforementioned databases, 66 overlapping target genes were found. The 66 overlapping target genes were derived from 52 compounds. Supplementary Table S3 listed the target genes that interact with the disease and chemicals. Gene ontology (GO) analysis and Kyoto Encyclopedia of Genes and Genomes (KEGG) enrichment analyses were conducted using the 66 overlapping target genes. Through GO analysis of the overlapping target genes, biological process, cellular component, and molecular function terms were identified (Figure 3A). Figure 3B shows the outcomes of functional annotation assessments of KEGG pathways. These pathways were connected to the endocrine system, signal transduction, and other biological processes in the human body. A chemical–gene–pathway network was created to clarify the complex interactions between potential active compounds, target genes, and KEGG pathways. As shown in Figure 4, nodes represented drugs, diseases, chemicals, target genes, and pathways, while edges represented the interaction of chemicals and target genes or the interaction of pathways and target genes. The network contained 130 nodes (52 chemicals, 66 target genes, and 10 pathways), along with 621 edges.

Some previous studies have reported that the chemical–gene–pathway interactions in a pharmacology network could be used to explore the potential mechanism underlying the effects of *Oleum Cinnamomi* [6,20]. However, owing to the small number of compounds selected in the previous literature, it was not possible to predict the correct signaling pathway. In this study, 52 detected compounds were used to construct the chemical–gene–pathway network, which could predict pathways more accurately. The screened compounds were also determined to be more suitable for *Oleum Cinnamomi* quality control. Ultimately, the visualized network was strongly linked to the effects of *Oleum Cinnamomi* on the disease and its potential active chemicals.

### 2.4. Selection of Index Components for the Quantitative Analysis of Oleum Cinnamomi

Three factors should be taken into account for TCM quality control: efficient and safe quality markers; standard reference with sufficient quantity; and quantitative approaches with broad applicability [21]. In the aforementioned network pharmacological analysis of *Oleum Cinnamomi*, 52 potential chemicals were found as potential active compounds. Considering these three aspects and operability, 10 compounds related to overlapping genes were selected as index components for quantitative analysis, for which the relative content was higher than 1% and standards could be purchased in the market. These compounds were as follows: β-pinene, sabinene, limonene, eucalyptol, *p*-cymene, terpinen-4-ol, 4-(1-methylethyl)-2-cyclohexen-1-one, α-terpineol, caryophyllene oxide, and guaiol.

In addition, these compounds were reported to have pharmacological effects. Studies have shown that β-pinene is a bioactive compound with antioxidant, cytoprotective, and neuroprotective effects [22,23]. Eucalyptol, the most abundant component of *Oleum Cinnamomi*, has promising potential for cardiovascular disease [24]. As a strong natural antioxidant substance, *p*-cymene has antioxidant activities and is effective for the treatment of some severe diseases, such as cancer, diabetes, and coronary heart disease [25]. Further, one study found that sabinene exhibits concentration-dependent antioxidant activity [26]. It was also reported that the other compounds have varying degrees of pharmacological effects on cardiovascular disease [27,28,29,30,31]. To further improve the quality control of *Oleum Cinnamomi*, 20 batches were selected to determine the contents of these 10 potential effective compounds.

### 2.5. Quantitative Analysis of Multiple Batches of Oleum Cinnamomi

#### 2.5.1. Method Validation

Based on the guidelines offered by the Chinese Pharmacopoeia [32], the created approach was validated. Specificity, linearity, lower limit of quantification (LLOQ), accuracy, precision, repeatability, stability, and recovery are all part of the methodological verification process. The inspection results showed that the solvent did not interfere with the determination of the 10 components. The linearity of the standard curve of the 10 compounds was greater than 0.999, indicating good linearity. The LLOQ is the lower limit at which an assay can provide quantitative results, and the RSD values for accuracy, precision, stability, and the recovery rate were all <3.0%. These results show that the method has good applicability. For the specific parameters of each part, see the Supplementary Material “Method validation” section in the Supporting Information.

#### 2.5.2. Assay Result

Ten components with source specificity or stronger activity than the tested components were selected by network pharmacology, and GC-MS SIM was used to determine their contents in 20 batches of *Oleum Cinnamomi* under the optimized conditions. It was preliminarily found that the contents of these 10 compounds differed in *Oleum Cinnamomi* from different origins. The results are presented in Supplementary Table S4.

### 2.6. Multivariate Statistical Analysis

#### 2.6.1. Hierarchical Clustering Analysis (HCA)

**Figure 5:**
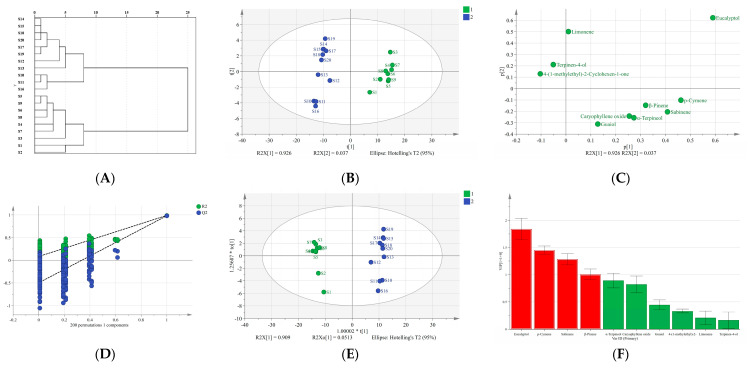
Multivariate statistical analysis of 10 potential effective compounds in 20 batches of *Oleum Cinnamomi*. Hierarchical cluster analysis dendrogram (**A**). Principal component analysis: score plot (**B**), loading plot (**C**). Orthogonal partial least squares-discriminant analysis (OPLS-DA): 200 response permutation tests for the OPLS-DA model (**D**), score plot (**E)**, and VIP plot (**F**).

The contents of 10 compounds in 20 batches of *Oleum Cinnamomi* were used as variables in SPSS 20.0, and HCA was carried out using the between-group linkage method with the Euclidean distance as the metric. As the dendrogram showed in Figure 5A, there were two types of samples in 20 batches when the Euclidean distance was 10 (I and II). According to the HCA results, the overall difference in *Oleum Cinnamomi* produced based on these batches was relatively significant, indicating that the quality of the essential oil was affected by the provenance of the original medicinal materials utilized in the preparation, the harvest season, and quality variations in the original medicinal materials between batches, among other factors.

#### 2.6.2. Principal Component Analysis (PCA)

The 20 × 10 data matrix was imported into SIMCA 14.1 software to perform PCA on the 20 batches of *Oleum Cinnamomi* in order to conduct a more thorough analysis of the data. The cumulative variance contribution rate of the first two principal components was 96.3%, which indicated that they could reflect the essential traits and the majority of the data of *Oleum Cinnamomi*. As shown in Figure 5B, 20 batches of samples were divided into two categories, approximately distributed on the left and right sides. This result further validated the classification results of HCA. According to the distance between the variable and the origin, the influence of the variable on the weight of the principal component was determined. The farther the point, the greater the weight of the influence of the variable on the principal component [33]. As shown in Figure 5C, except for four variables (eucalyptol, *p*-cymene, sabinene, and β-pinene), other variables were not dominant in terms of the significant components, indicating that these four variables might be the reason for the offset in the distribution. Based on the distribution of these four variables, eucalyptol made the largest contribution to principal component 1 and 2, whereas *p*-cymene, sabinene, and β-pinene had greater contributions to principal component 1.

#### 2.6.3. Orthogonal Partial Least Squares Discriminant Analysis (OPLS-DA)

**Figure 6:**
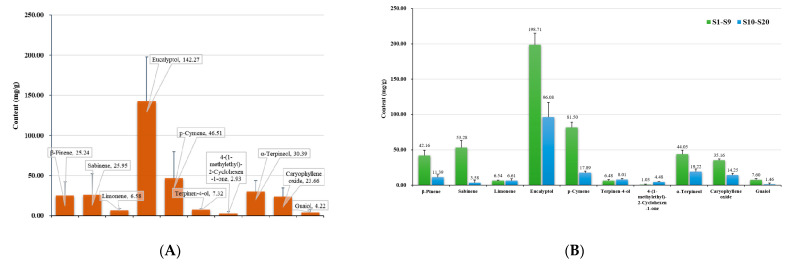
Average contents of 10 ingredients in 20 batches (**A**), and average contents of the 10 ingredients in S1–S9 and S10–S20 (**B**).

Based on the PCA, the 20 batches of samples were subjected to OPLS-DA. *R*^2^ and *Q*^2^ were both greater than 0.5, demonstrating the good predictive ability of this model [34]. In addition, 200 response ranking tests were performed to determine whether the generated model was related to an over-fitting phenomenon. As a result, all *R*^2^ and *Q*^2^ values calculated by random sorting were less than the original values, and the *Q*^2^ regression line showed a negative intercept with the *Y-axis* (Figure 5D). These results indicated that the model was effective and that overfitting did not take place [35]. Therefore, it could be used to screen differential quality markers in *Oleum Cinnamomi*. As shown in Figure 5E, the 20 batches of samples were divided into two categories, which was consistent with the classification results of HCA and PCA. To further explore the elements that contributed to variations among the 20 batches of samples, we extracted the variable importance in projection (VIP) value of 10 variables from the OPLS-DA model, with VIP > 1 as the benchmark [36]. As shown in Figure 5F, eucalyptol, *p*-cymene, sabinene, and β-pinene were suggested to be the main reasons for the difference. Meanwhile, the average value of components in the two categories (namely S1–S9 and S10–S20) could roughly be used to explain that eucalyptol, *p*-cymene, sabinene, and β-pinene were the difference-associated compounds (Figure 6). These four compounds could be considered to have a marker effect, which were consistent with the importance weight variables found in the PCA loading plot.

*Oleum Cinnamomi* is the essential oil obtained from *Fructus Cinnamomi*, which is the dry and ripe fruit of *Cinnamomum migao*, a large evergreen of the Lauraceae family [37]. *C. migao* is only distributed in the dry and hot valleys of the Karma-Fenglin Mountain region. Numerous soluble rocks on the inner surface are exposed, and some insoluble rocks are exposed locally, resulting in the dispersion of non-karst landforms across the region [38]. This unique geographical distribution pattern forms a heterogeneous microenvironment, profoundly affecting the species distribution in the region [39]. Meanwhile, a study found that the climate differences in this region promote differentiation of the *C. migao* fungal community. Fungal groups stimulate the plant body to produce secondary metabolites, which are often the medically active ingredients of medicinal plants. According to a report, sabinene, the secondary metabolites of saprophytic fungi, can reflect the quality of *C. migao* in different regions [37]. In this study, the content of quality markers in Luodian samples was much higher than those in other origins. We inferred that the high average altitude (about 800m) and warm climate of Luodian may led to an increase number of saprophytic fungi. It can be preliminarily considered that the *Oleum Cinnamomi* prepared from *C. migao* in Luodian may have better effects. Therefore, during the industrial production process for such preparations, the source and quality of eucalyptol, *p*-cymene, sabinene, and β-pinene should be monitored. As the literature has reported, these compounds have good curative effects with respect to the treatment of coronary heart disease [24,25], which is the indication of *Oleum Cinnamomi*. In order to further improve the stability of preparation quality of *Oleum Cinnamom*, the control of the aforementioned components could be increased through the follow-up quality research. 

## 3. Materials and Methods

### 3.1. Reagents and Materials

In total, 20 batches of herbs (S1–S11, Guizhou, China; S12–S15, Yunnan, China; S16–S20, Guangxi, China) were procured from Guizhou Yibai Pharmaceutical Ltd. The medicinal materials were determined to be authentic by Associate Professor Liu Chunhua, at the Department of Pharmacognosy, School of Pharmaceutical, Guizhou Medical University. Sample information for the 20 batches of *Fructus Cinnamomi* is presented in Supplementary Table S5.

The standards for eucalyptol (Lot. AF20032351), sabinene (Lot. AF20122004), terpinen-4-ol (Lot. AF21032106), β-pinene (Lot. AF21072701), α-terpineol (Lot. AF20041531), and *p*-cymene (Lot. AF21022701) were purchased from Chengdu Alfa Biotech (Chengdu, China). The reference substance for guaiol (Lot. ZZS-20-H219-A1) was purchased from Shanghai Zhenzhun Science & Technology Co., Ltd (Shanghai, China), whereas the standard for 4-(1-methylethyl)-2-cyclohexen-1-one (Lot. 2-DRR-35-1) was obtained from Toronto Research Chemicals (Toronto, ON, Canada). The naphthalene standard (Lot. 21050063) was obtained from the Tan Mo Quality Inspection Standard Material Center (Changzhou, China). The standards for caryophyllene oxide (Lot. CFS202002), limonene (Lot. MK181216-03), and cyclohexanone (Lot. GF201410-20) were prepared at the Beijing Northern Weiye Metrology Technology Research Institute (Beijing, China). Distilled water was purchased from Guangzhou Watsons Food and Beverage (Guangzhou, China). All other chemicals were of analytical reagent grade. The purity of all standards was greater than 98%.

### 3.2. Extraction of Oleum Cinnamomi

According to the extraction principles of the Pharmacopoeia of the People’s Republic of China [32], *Oleum Cinnamomi* was extracted. Approximately 20.0 g of *Fructus Cinnamomi* powder (60 mesh) was accurately weighed and soaked with 240 mL water for 0.5 h before extraction. After extracting it for 5 h, an appropriate amount of anhydrous sodium sulfate was added, the sample was subsequently sealed overnight, and it was centrifuged at 8000 r/min for 10 min the next day to collect the upper oil layer. The oil yield was 11.30%.

### 3.3. Preparation of the Standard Solution and Sample Solution

Samples of 10 standard compounds were accurately weighed, placed into a volumetric bottle, dissolved in ethyl acetate, and brought to a specific volume. The concentrations of the 10 standard compounds in the mixed standard solutions were as follows: 1.586 mg/mL, β-pinene; 1.400 mg/mL, sabinene; 1.003 mg/mL, limonene; 8.820 mg/mL, eucalyptol; 5.380 mg/mL, *p*-cymene; 1.660 mg/mL, terpinen-4-ol; 1.654 mg/mL, caryophyllene oxide; 1.538 mg/mL, 4-(1-methylethyl)-2-cyclohexen-1-one; 8.450 mg/mL, α-terpineol; 1.650 mg/mL, guaiol. In addition, the stock solutions of cyclohexanone and naphthalene internal standards were prepared by dissolving the standard references with ethyl acetate and finally making the concentration reach a level of 3.300 mg/mL and 2.710 mg/mL, respectively. An appropriate volume of the aforementioned internal standard solution was accurately transferred to a 100 mL volumetric flask and diluted to the mark with ethyl acetate. The final concentrations of cyclohexanone and naphthalene were 150 μg/mL and 3 μg/mL, respectively.

The essential oil (50 mg) was accurately weighed from the S9 batch of herbs into a 50 mL volumetric flask. Ethyl acetate was added at a constant volume, and the sample was shaken. After 1.0 mL of this solution was transferred to another 50 mL volumetric flask, ethyl acetate was added to the flask at a constant volume, and the sample was shaken and mixed to obtain the test solution for identifying chemical components in *Oleum Cinnamomi*. In addition, 100 mg of essential oil was accurately weighed from 20 batches of the herbs into a 20 mL volumetric flask. Ethyl acetate was added to the oil at a constant volume, and the sample was shaken. After 1.0 mL of this solution was transferred to another 50 mL volumetric flask, 1.0 mL of cyclohexanone and naphthalene internal standard solution and a constant volume of ethyl acetate were added. The test solutions were obtained after shaking the aforementioned solution well, and these were used to detect the contents of 10 potentially effective compounds screened by the network pharmacological analysis of *Oleum Cinnamomi*. All solutions were stored at −20°C for further study.

### 3.4. Chromatographic and Mass Spectrometric Conditions for Qualitative and Quantitative Analysis

A Shimadzu GC-MS-TQ8050NX (Shimadzu Co., Ltd., Kyoto, Japan) was used for specimen analysis. Chromatographic separation of the analytes was performed in an SH-Stabilwax capillary column (30 m × 0.32 mm, 0.25 μm film thickness). For qualitative analysis, the injector was operated in splitless mode with an injection volume of 1.0 μL, and the injector port was maintained at 220 °C. The column temperature program is shown in Table 2. For quantitative analysis, except that the split ratio was 50:1, the rest of the analysis conditions were the same as those of the qualitative analysis.

Mass spectrometry conditions were as follows: detection voltage, 0.25 kV; ion source temperature, 200 °C; interface temperature, 230 °C; electron impact ionization mode was used for ionization; mass range, 30–400 amu; SCAN mode was used for qualitative analysis, whereas SIM mode was used for quantitative analysis. Ten potential active components and internal standards mass spectrometry conditions are shown in Supplementary Table S6. Data analysis was performed using the Shimadzu GC-MS solution software (Shimadzu Co., Ltd., Japan).

### 3.5. Network Pharmacological Analysis of Oleum Cinnamomi

According to that mentioned in the related literature [40,41,42], potential active chemicals were selected from the identified compounds in Section 2.4 by GC-MS, and their molecular structures were confirmed using PubChem [43]. Target genes (probability > 0) associated with the screening chemicals were obtained from SwissTarget Prediction [44], only ‘*Homo sapiens*’ targets were selected, and the chemical–target gene database was built. Information on potential target genes associated with coronary heart disease was found in the Therapeutic Target Database [45], OMIM [46], DisGeNet [47] (score > 0.1), and GeneCards [48], and only ‘*Homo sapiens*’ targets were selected. Further, the target name, ID, and organism were confirmed with UniProt [49], and then, the disease–target gene database was built. Afterwards, chemical target genes and disease target genes were compared to obtain the intersecting target genes, which were imported into *R* software for GO and KEGG enrichment analyses. Next, the pathway analysis was visualized using Cytoscape [50]. Based on these methods, a “drug-active ingredient-target-pathway-disease” network was constructed. In addition, compounds associated with intersecting target genes were selected as potential active ingredients for quantitative analysis.

### 3.6. Statistical Analysis

The relative percent content of each component was calculated by the peak area normalization method for the qualitative analysis of *Oleum Cinnamomi*. Some important variables screened by network pharmacology that made major contributions to the diversity of different samples were screened and used as the markers for further *Oleum Cinnamomi* quality control. Thus, a quick and simple method was established to select unqualified medicines, which would improve work efficiency. Different batches of *Oleum Cinnamomi* were analyzed using SPSS software (20.0; IBM, Chicago, IL, USA) and SIMCA software (14.1; Umetrics, Malmö, Scania, Sweden).

## 4. Conclusions

In this study, an efficient method was developed to identify the 57 chemical components of *Oleum Cinnamomi*. In addition, a network pharmacology strategy was successfully applied for the selection of potential active chemical components of *Oleum Cinnamomi*. Meanwhile, based on the qualitative analysis protocol, a sensitive and specific method was established to enable quantitative analysis of the 10 potential active chemical components of *Oleum Cinnamomi*. The novel method was confirmed to be authentic and stable, and it was used to analyze 20 batches of *Oleum Cinnamomi*. Multivariate statistical analysis methods (HCA, PCA, and OPLS-DA) were then used to classify and evaluate different batches of *Oleum Cinnamomi*. Eucalyptol, *p*-cymene, sabinene, and β-pinene were found to affect the stability of the essential oil. This method was used to analyze 20 batches of *Oleum Cinnamomi*, and a temporary content standard was established for the quality control of *Oleum Cinnamomi*. By this method, compounds with activity and quality difference were screened out as quality markers for *Oleum Cinnamomi*. The strategy developed for the quality control of *Oleum Cinnamomi*, assisted by a network pharmacology strategy and multivariate statistical analysis, could also be applied to other TCMs.

## Figures and Tables

**Table 1:** Chemical constituents of *Oleum Cinnamomi*.

NO.	Chemical Constituents	Molecular Formula	Molecular Weight	Relative Percentage (%)
1 *	β-Pinene	C_10_H_16_	136	3.53
2 *	Sabinene	C_10_H_16_	136	2.53
3	α-Phellandrene	C_10_H_16_	136	0.95
4 *	β-Myrcene	C_10_H_16_	136	0.49
5	Terpinolene	C_10_H_16_	136	0.25
6	2,3-Dehydro-1,8-cineole	C_10_H_16_O	152	0.23
7 *	Limonene	C_10_H_16_	136	1.43
8 *	Eucalyptol	C_10_H_18_O	154	25.03
9 *	γ-Terpinene	C_10_H_16_	136	0.51
10 *	*p*-Cymene	C_10_H_14_	134	4.66
11 *	4-Carene	C_10_H_16_	136	0.19
12 *	2-Nonanone	C_9_H_18_O	142	0.24
13	Copaene	C_15_H_24_	204	0.83
14 *	4-Thujanol	C_10_H_18_O	154	0.41
15	Pinocarvone	C_10_H_14_O	150	0.41
16 *	Borneol acetate	C_12_H_20_O_2_	196	0.28
17 *	Caryophyllene	C_15_H_24_	204	0.77
18 *	Terpinen-4-ol	C_10_H_18_O	154	1.70
19	Dodecane, 2-methyl-	C_13_H_28_	184	0.18
20 *	Myrtenal	C_10_H_14_O	150	0.45
21	Butanedioic acid, 2,3-bis(acetyloxy)-	C_8_H_10_O_8_	234	0.22
22	Cyclohexanol, 4-(1-methylethyl)-	C_9_H_18_O	142	0.38
23	6,6-dimethyl-2-methylidenebicyclo[3.1.1]heptan-3-ol	C_10_H_16_O	152	0.90
24 *	4-(1-methylethyl)-2-Cyclohexen-1-one	C_9_H_14_O	138	1.68
25	Cyclohexanemethanol, α, α- dimethyl-4-methylene-	C_10_H_18_O	154	0.66
26	Verbenol	C_10_H_16_O	152	0.58
27	4a,8-Dimethyl-2-(prop-1-en-2-yl)-1,2,3,4,4a,5,6,7-octahydronaphthalene	C_15_H_24_	204	0.33
28 *	α-Terpineol	C_10_H_18_O	154	6.23
29	2,2,5a,9-Tetramethyloctahydro-2*H*-3,9a-methanobenzo[b]oxepine	C_15_H_26_O	222	1.02
30	1-Cyclohexene-1-carboxaldehyde, 4-(1-methylethyl)-	C_10_H_16_O	152	0.39
31	β-cadinene	C_15_H_24_	204	2.60
32*	Cuminaldehyde	C_10_H_12_O	148	0.76
33*	Myrtenol	C_10_H_16_O	152	0.51
34	2*H*-3,9a-Methano-1-benzoxepin, octahydro-2,2,5a,9-tetramethyl-	C_15_H_26_O	222	0.60
35	2-Cyclohexen-1-ol, 2-methyl-5-(1-methylethenyl)-	C_10_H_16_O	152	0.36
36 *	2-(4-Methylphenyl)propan-2-ol	C_10_H_14_O	150	0.54
37	4-Epicubebol	C_15_H_26_O	222	0.18
38	α-Calacorene	C_15_H_20_	200	0.57
39	Cubebol	C_15_H_26_O	222	0.26
40 *	Caryophyllene oxide	C_15_H_24_O	220	1.30
41	1,3-Cyclohexadiene-1-methanol, 4-(1-methylethyl)-	C_10_H_16_O	152	0.3
42	1,5,5,8-Tetramethyl- 12- oxabicyclo[9.1.0]dodeca-3,7-diene	C_15_H_24_O	220	0.55
43	1,4-Cyclohexadiene-1-methanol, 4-(1-methylethyl)-	C_10_H_16_O	152	0.38
44	Cyclohexanemethanol, 4-ethenyl-α,α,4-trimethyl-3- (1-methylethenyl)-	C_15_H_26_O	222	1.26
45 *	Guaiol	C_15_H_26_O	222	1.17
46	Rosifoliol	C_15_H_26_O	222	2.48
47	2-Naphthalenemethanol, 1,2,3,4,4a,5,6,7- octahydro- α,α, 4a,8-tetramethyl-	C_15_H_26_O	222	1.96
48	1*H*-Cycloprop[e]azulen-7-ol, decahydro-1, 1,7- trimethyl- 4-methylene-, [1ar-(1aα,4aα,7β,7aβ,7bα)]-	C_15_H_24_O	220	1.07
49	4a,8-Dimethyl-4,4a,5,6,7,8-hexahydronaphthalen-2(3*H*)-one	C_12_H_18_O	178	0.56
50	1,7-di-iso-propylnaphthalene	C_16_H_2_0	212	0.49
51	1,3-di-iso-propylnaphthalene	C_16_H_20_	212	0.35
52	5-Azulenemethanol, 1,2,3,3a,4,5,6,7-octahydro- α,α, 3,8-tetramethyl-	C_15_H_26_O	222	0.39
53	Agarospirol	C_15_H_26_O	222	3.59
54	1,4-di-iso-propylnaphthalene	C_16_H_20_	212	0.23
55	*n*-Decanoic acid	C_10_H_20_O_2_	172	0.93
56	2,4-Di-tert-butylphenol	C_14_H_22_O	206	1.97
57	Dodecanoic acid	C_12_H_24_O_2_	200	0.23
Total (%)				83.05

* These compounds were confirmed by comparison with the standard compounds.

**Table 2:** Column temperature program.

Rate (°C/min)	Temperature (°C)	Retention Time (min)
-	40	5
2	50	-
10	100	-
5	150	-
2	190	1
50	230	3

## Data Availability

The authors declare that all data supporting the findings of this study are available within the article.

## Supplementary Materials

The following supporting information can be downloaded at: https://www.mdpi.com/article/10.3390/molecules27196391/s1. Method Validation: Figure sMV1. GC-MS SIM chromatogram of reference solution (A), sample solution (B) and blank control solution (C). Table sMV1. The calibration curve, LLOQ, accuracy, precision, reproducibility and stability of 10 components in *Oleum Cinnamomi*. Table sMV2. Sample recovery test of 10 components in *Oleum Cinnamomi*. Screening of Extraction Methods. Figure sSEM1. Effect of crushing particle size on oil yield of *Oleum Cinnamomi*. Figure sSEM2. Effect of solid-liquid ratio on oil yield of *Oleum Cinnamomi*. Figure sSEM3. Effect of soaking time on oil yield of *Oleum Cinnamomi*. Figure sSEM4. Effect of extraction time on oil yield of *Oleum Cinnamomi*. Tables and Figures. Table S1: Chemical-target gene database. Table S2: Disease-target gene database. Table S3: Target genes interacting with both chemicals and coronary heart disease. Table S4: The content of 10 ingredients in 20 batches of *Oleum Cinnamomi* (mg/g, *n*=3). Table S5: Sample information of *Fructus Cinnamomi*. Table S6: Ion information of multi-index components in *Oleum Cinnamomi*. Figure S1: GC-MS SIM chromatogram of reference solution (A), sample solution (B) and blank control solution (C).
