# Supplementary material for: Quality Control of Oleum Cinnamomi Assisted by Network Pharmacology Strategy
Source: Molecules. 2022 Sep 27;27(19):6391. doi: 10.3390/molecules27196391 (PMC9573265; doi:10.3390/molecules27196391)
Supplement: Supplementary file 1 [file molecules-27-06391-s001.zip › Supplementary material_Method validation.pdf]

Method validation

Specificity

The specificity of this method was studied. Precisely draw 1.0 μL of mixed reference solution and test solution respectively, and then inject them separately. The result was shown in Figure sMV1. The retention time of the test compound in the test solution is the same as the retention time of the 10 reference substances. Comparing the spectra of the measured component and the reference substance, the two were consistent, and there was no interference in the blank solvent, indicating that the method had good specificity.

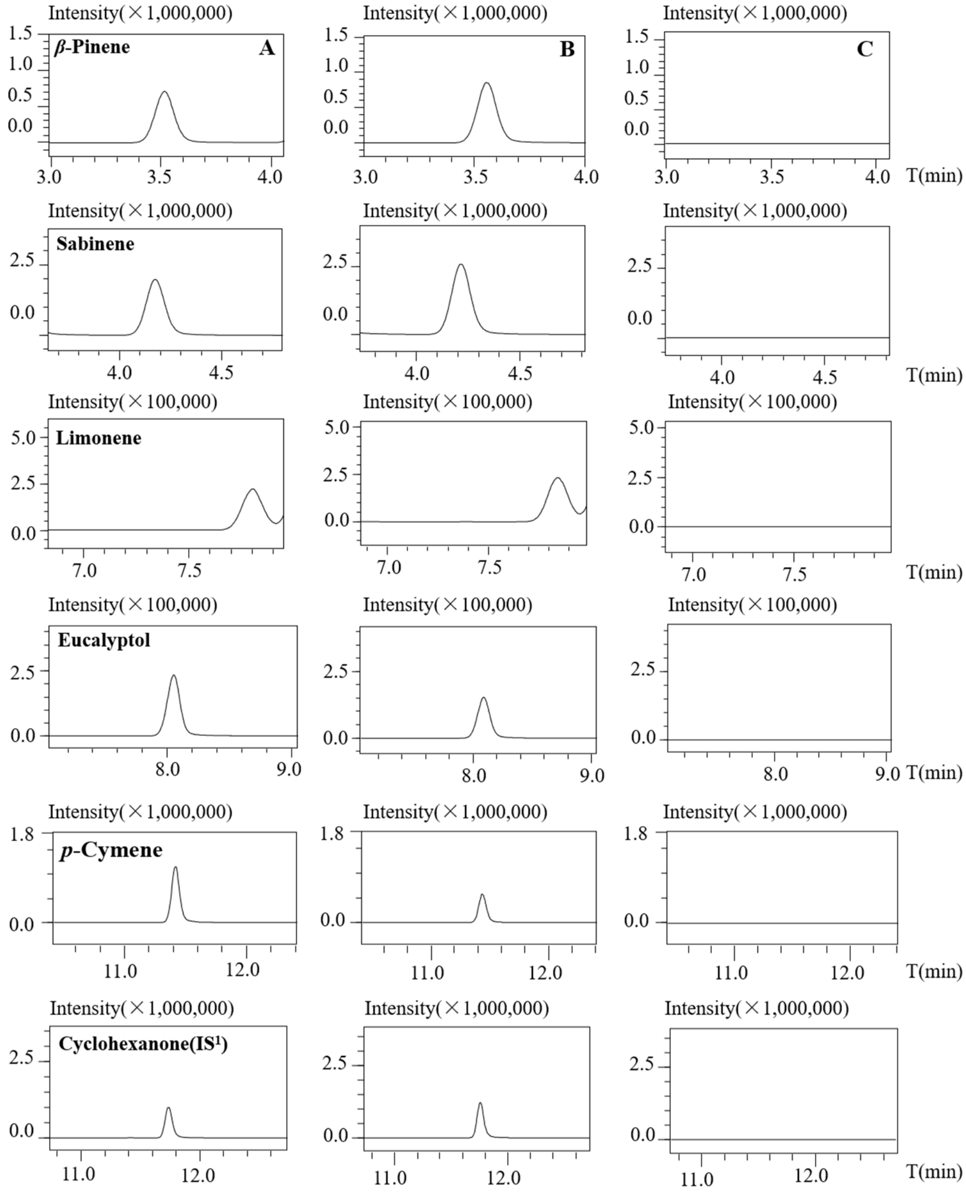

Figure sMV1. GC-MS SIM chromatogram of reference solution (A), sample solution (B) and blank control solution (C)

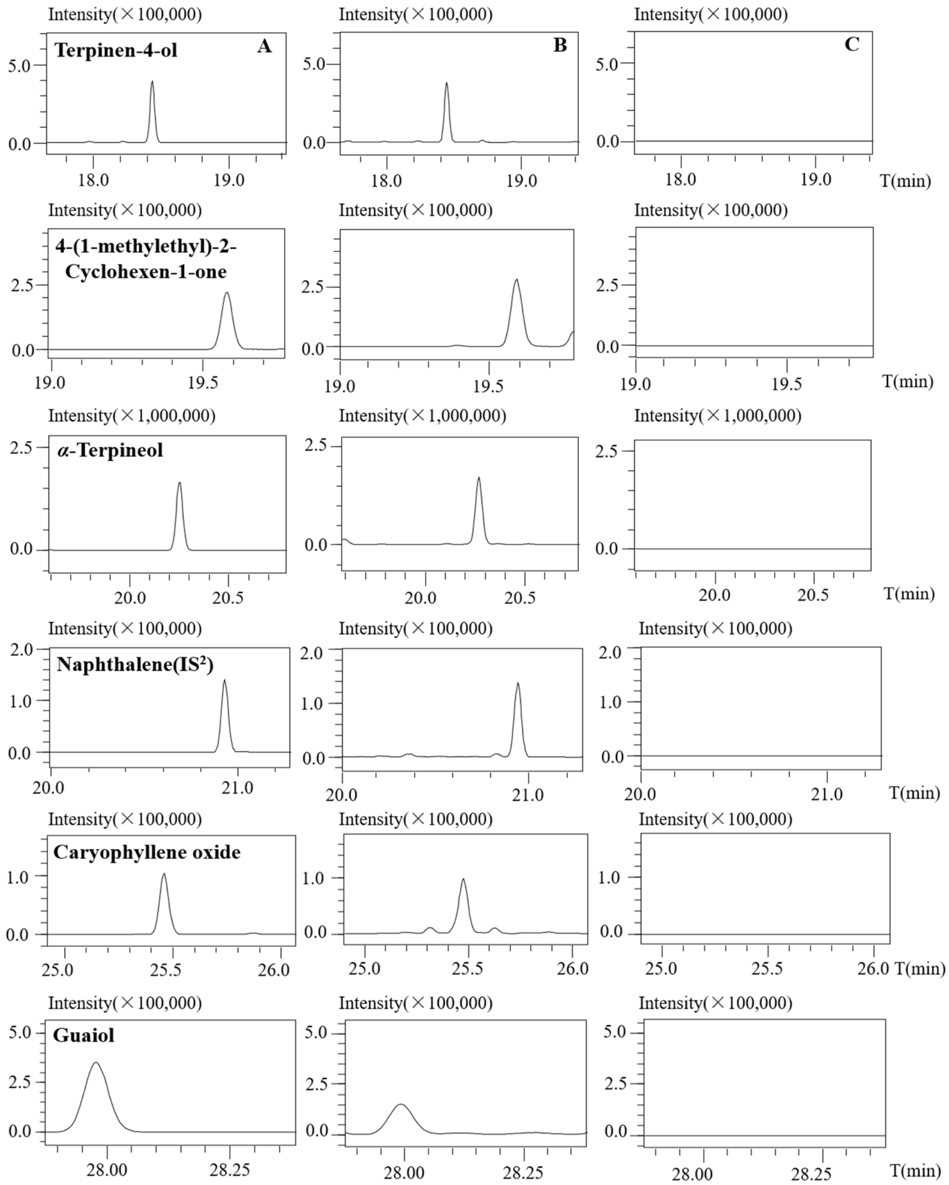

Figure sMV1(continued). GC-MS SIM chromatogram of reference solution (A), sample solution (B) and blank control solution (C)

Linearity and LOQ

At least six concentrations of 10 analyte solutions were analyzed, and then a calibration curve was drawn by plotting the relationship between the peak area and the concentration of each analyte. The calculation results were shown in Table sMV1. All analytes showed good linearity in a relatively wide concentration range. The working solution of the analyte is further diluted with ethyl acetate to produce a series of appropriate concentrations. Table sMV1 also lists the lower limit of quantification (LLOQ) for each compound studied.

Table sMV1. The calibration curve, LLOQ, accuracy, precision, reproducibility and stability of 10 components in *Oleum Cinnamomi*

No.	Analytes	Regression equation $y = Ax + B$	R^2	Liner Range (μg/mL)	LLOQ (μg/mL)	Precision (RSD, %, n=6)		Reproducibility (RSD, %, n=6)	Stability (RSD, %, n=7)
						Intra-day	Inter-day		
1	*β*-Pinene	y = 0.0297x + 0.0030	0.9999	0.79-79.30	0.79	0.64	1.92	1.23	1.67
2	Sabinene	y = 0.0850x - 0.0147	0.9999	0.17-84.00	0.17	1.76	1.51	1.60	1.54
3	Limonene	y = 0.0562x - 0.0037	0.9999	0.30-30.09	0.30	1.58	2.50	2.26	2.47
4	Eucalyptol	y = 0.0208x + 0.0057	1.0000	4.41-441.00	4.41	2.65	1.99	2.22	1.74
5	*p*-Cymene	y = 0.0287x + 0.0065	0.9999	2.02-201.75	2.02	1.18	2.21	2.49	2.23
6	Terpinen-4-ol	y = 0.0619x - 0.0008	1.0000	0.17-16.60	0.17	2.22	2.69	1.92	1.72
7	4-(1-methylethyl)-2-Cyclohexen-1-one	y = 0.8763x - 0.0176	1.0000	0.12-11.54	0.12	2.25	2.47	1.46	1.84
8	*α*-Terpineol	y = 0.0332x - 0.0024	1.0000	0.63-63.38	0.63	2.30	2.61	2.26	1.59
9	Caryophyllene oxide	y = 0.2813x - 0.0443	0.9999	0.50-49.62	0.50	2.13	2.17	2.01	2.05
10	Guaiol	y = 1.1139x - 0.1072	0.9997	0.04-20.63	0.04	1.79	2.05	2.47	2.63

Note: 1) *y*, concerntration (μg/mL); *x*, Area. 2) LLOQ, Lower Limit of quantification. 3) RSD, Relative standard deviation

Precision and reproducibility

By repeating the analysis of the known concentrations of 10 analytes five times a day, and repeating the experiment for three consecutive days, the intra-day and inter-day precision could be determined. To confirm the reproducibility, five different working solutions prepared from the same sample (batch: S9) were analyzed. Relative standard deviation (R.S.D.) was used as a measure of precision and reproducibility, and the results were shown in Table sMV1. The intra-day precision, inter-day precision, and reproducibility R.S.D. values of the 10 compounds in the table were all less than 3.0%, and the developed method had good reproducibility.

Stability

The stability of the sample solution was test at room temperature. The analysis of the sample solution was repeated at 0, 2, 4, 6, 8, 12, 24 h. The results were shown in Table sMV1. During the test, the analytes was found to be very stable within 24 hours (1.67% ≤ R.S.D. ≤ 3.0%).

Recovery

A recovery test was performed to further study the accuracy of the method by equalizing the concentration level of the mixed standard solution with a known amount of *Oleum Cinnamomi*. The resulting sample was then extracted and analyzed by the method described. The average

recovery rate was evaluated by calculating the ratio of the detected amount to the added amount. The recovery rate of this method was in the range of 92.13-104.98%, RSD represented the result, as shown in Table sMV2, which was less than 4.0%, indicating the method was considered accurate.

Table sMV2. Sample recovery test of 10 components in *Oleum Cinnamomi*

No.	Analysts	Sample weigh (g)	Content (mg)	Skipped (mg)	Found (mg)	Recovery (%)	Mean (%)	RSD (%)
1	*β*-Pinene	0.0540	1.675	1.717	3.487	105.522	102.27	2.71
		0.0511	1.585	1.717	3.390	105.092		
		0.0509	1.579	1.717	3.270	98.489		
		0.0518	1.607	1.717	3.364	102.368		
		0.0523	1.622	1.717	3.338	99.930		
		0.0514	1.594	1.717	3.349	102.204		
2	Sabinene	0.0540	1.603	1.760	3.275	94.992	98.27	2.19
		0.0511	1.517	1.760	3.242	98.006		
		0.0509	1.511	1.760	3.283	100.708		
		0.0518	1.537	1.760	3.284	99.261		
		0.0523	1.552	1.760	3.254	96.697		
		0.0514	1.526	1.760	3.285	99.960		
3	Limonene	0.0540	0.773	0.802	1.575	99.933	99.31	1.79
		0.0511	0.731	0.802	1.515	97.692		
		0.0509	0.729	0.802	1.522	98.912		
		0.0518	0.741	0.802	1.552	101.020		
		0.0523	0.749	0.802	1.562	101.361		
		0.0514	0.736	0.802	1.514	96.967		
4	Eucalyptol	0.0540	10.160	9.702	20.402	105.564	104.15	1.89
		0.0511	9.614	9.702	19.757	104.543		
		0.0509	9.577	9.702	19.852	105.909		
		0.0518	9.746	9.702	19.783	103.452		
		0.0523	9.840	9.702	19.593	100.524		
		0.0514	9.671	9.702	19.849	104.911		
5	*p*-Cymene	0.0540	1.845	1.705	3.640	105.261	103.41	2.21
		0.0511	1.746	1.705	3.554	106.037		
		0.0509	1.739	1.705	3.480	102.055		
		0.0518	1.770	1.705	3.537	103.621		
		0.0523	1.787	1.705	3.487	99.687		
		0.0514	1.756	1.705	3.526	103.786		
6	Terpinen-4-ol	0.0540	0.519	0.498	1.029	102.478	104.98	2.46
		0.0511	0.491	0.498	0.999	101.955		
		0.0509	0.489	0.498	1.019	106.452		
		0.0518	0.498	0.498	1.014	103.712		
		0.0523	0.503	0.498	1.039	107.767		
		0.0514	0.494	0.498	1.029	107.496		
7	4-(1-methylethyl)-2-	0.0540	0.431	0.400	0.838	101.640	102.49	1.08

	Cyclohexen-1-one	0.0511	0.408	0.400	0.819	102.665		
		0.0509	0.406	0.400	0.817	102.571		
		0.0518	0.414	0.400	0.831	104.437		
		0.0523	0.418	0.400	0.822	101.226		
		0.0514	0.410	0.400	0.820	102.386		
		0.0540	2.453	2.535	5.017	101.143		
		0.0511	2.321	2.535	4.921	102.572		
8	α-Terpineol	0.0509	2.312	2.535	4.829	99.318	98.58	2.92
		0.0518	2.353	2.535	4.776	95.588		
		0.0523	2.375	2.535	4.817	96.318		
		0.0514	2.334	2.535	4.781	96.515		
		0.0540	0.669	0.620	1.322	105.300		
		0.0511	0.633	0.620	1.284	104.988		
9	Caryophyllene oxide	0.0509	0.630	0.620	1.271	103.312	104.82	1.83
		0.0518	0.642	0.620	1.275	102.161		
		0.0523	0.648	0.620	1.301	105.389		
		0.0514	0.637	0.620	1.305	107.744		
		0.0540	0.499	0.462	0.932	93.831		
		0.0511	0.472	0.462	0.929	99.052		
10	Guaiol	0.0509	0.470	0.462	0.923	98.031	95.49	2.60
		0.0518	0.478	0.462	0.917	95.055		
		0.0523	0.483	0.462	0.917	94.038		
		0.0514	0.475	0.462	0.904	92.929		
