# Supplementary material for: Quality Control of Oleum Cinnamomi Assisted by Network Pharmacology Strategy
Source: Molecules. 2022 Sep 27;27(19):6391. doi: 10.3390/molecules27196391 (PMC9573265; doi:10.3390/molecules27196391)
Supplement: Supplementary file 1 [file molecules-27-06391-s001.zip › Supplementary material_Screening of Extraction Methods.pdf]

Screening of Extraction Methods

Calculation method of oil yield

The formula for calculating the oil yield is as follows:

$$\text{Oil yield (\%)} = \frac{\text{Weight of volatile oil}}{\text{Weight of } \textit{Oleum Cinnamomi}} \times 100\%$$

Crushing particle size

After *Oleum Cinnamomi* was pulverized, different kinds of particle size (10, 30, 60, 80, 100 mesh) were investigated. Approximately 20.0 g of powder was accurately weighed and soaked with 240 mL water. After extracting for 5 h, an appropriate amount anhydrous sodium sulfate was added, subsequently was sealed overnight, and was centrifuged at 8000 r/min for 10 min in the next day to collect the upper oil layer. The oil yield was calculated. As shown in Figure sSEM1, when the particle size is 10-60 mesh, the oil yield of *Oleum Cinnamomi* is increased with the increasing of particle size, and when the particle size reaches 60 mesh or more, the oil yield of volatile oil decreases. Therefore, it is appropriate to choose a 60-mesh pulverized particle size.

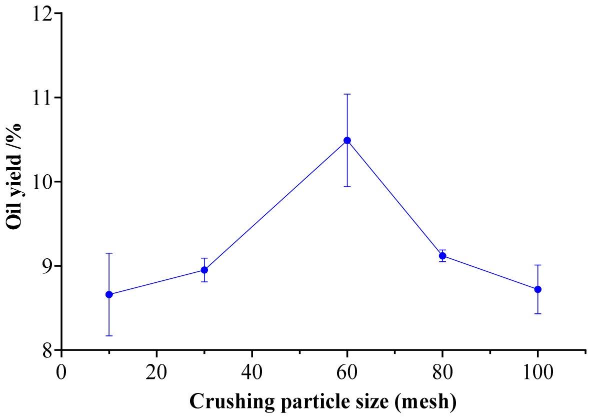

Figure sSEM1. Effect of crushing particle size on oil yield of *Oleum Cinnamomi*

Solid-liquid ratio

After *Oleum Cinnamomi* was pulverized, different kinds of solid-liquid ratio (1:6, 1:8, 1:10, 1:12, 1:14 g/mL) were investigated. Approximately 20.0 g of powder (60 mesh) was accurately weighed and soaked with different proportions of water. After extracting for 5 h, an appropriate amount anhydrous sodium sulfate was added, subsequently was sealed overnight, and was centrifuged at 8000 r/min for 10 min in the next day to collect the upper oil layer. The oil yield was calculated. As shown in Figure sSEM2, with the increase of the solid-liquid ratio, the oil yield first increased and then decreased. When the solid-liquid ratio was 1:8 (g/mL), the oil yield of *Oleum Cinnamomi* was the highest, so the solid-liquid ratio of 1:8 (g/mL) was selected.

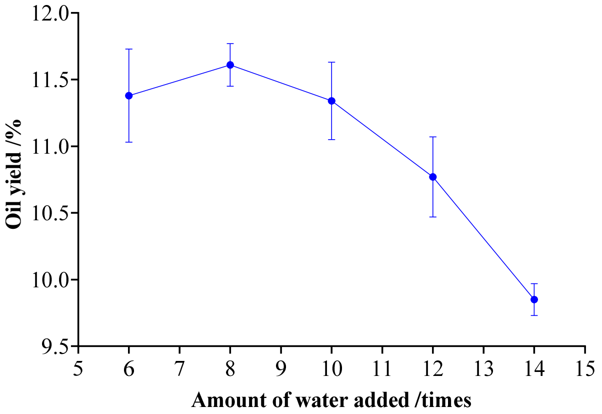

Figure sSEM2. Effect of solid-liquid ratio on oil yield of *Oleum Cinnamomi*

Soaking time

After *Oleum Cinnamomi* was pulverized, different kinds of soaking time (0, 0.5, 1, 1.5, 2 h) were investigated. Approximately 20.0 g of powder (60 mesh) was accurately weighed and soaked with 240 mL water for different lengths of time before extraction. After extracting for 5 h, an appropriate amount anhydrous sodium sulfate was added, subsequently was sealed overnight, and was centrifuged at 8000 r/min for 10 min in the next day to collect the upper oil layer. The oil yield was calculated. As shown in Figure sSEM3, with the prolongation of soaking time, the oil yield of *Oleum Cinnamomi* reached the maximum value when the soaking time was 0.5 h. Therefore, 0.5 h of soaking time was chosen.

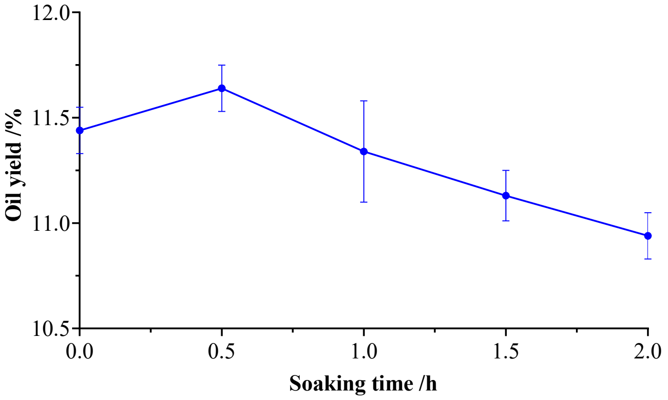

Figure sSEM3. Effect of soaking time on oil yield of *Oleum Cinnamomi*

Extraction time

After *Oleum Cinnamomi* was pulverized, different kinds of extraction time (2, 3, 4, 5, 6 h) were investigated. Approximately 20.0 g of powder (60 mesh) was accurately weighed and soaked with 240 mL water before extraction. After extracting for different lengths of time, an appropriate amount anhydrous sodium sulfate was added, subsequently was sealed overnight, and was centrifuged at 8000 r/min for 10 min in the next day to collect the upper oil layer. The oil yield was calculated. As shown in Figure sSEM4, with the prolongation of extraction time, the oil yield of *Oleum Cinnamomi* showed a trend of rising and then slowly decreasing, and reached highest at 5 h. Therefore, 5 h of extraction time was chosen.

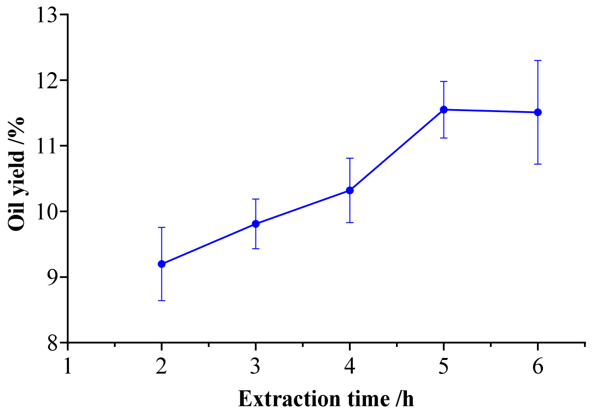

Figure sSEM4. Effect of extraction time on oil yield of *Oleum Cinnamomi*
