# Supplementary material for: Quality Control of Oleum Cinnamomi Assisted by Network Pharmacology Strategy
Source: Molecules. 2022 Sep 27;27(19):6391. doi: 10.3390/molecules27196391 (PMC9573265; doi:10.3390/molecules27196391)
Supplement: Supplementary file 1 [file molecules-27-06391-s001.zip › Supplementary material_Tables and Figures.pdf]

Table S1. Chemical-target gene database

No.	Chemical constituents	Target gene
1	β-Pinene	SLC5A7/HSD11B1/UGT2B7/PPARA/CNR2/PTGS1/NR1H3/ACHE/SHBG/PTPN1/NR1I3/CYP19A1/ESR1/CHRM2/SLC6A2/SLC6A4/CYP2C19/AR/FAAH/TRPV1
2	Sabinene	HSD11B1/UGT2B7/PPARA/CNR2/SLC5A7/NR1H3/ACHE/CYP19A1/ESR1/CHRM2/PTGS1/SLC6A2/SLC6A4/CYP2C19/AR/NR1H4/SAE1/UBA2/SHBG/PTGES/AKR1B10/POLB/PTPN1/NR1I3
3	α-Phellandrene	PPARA/CNR2/ADORA1/ADORA2A/ADORA3
4	β-Myrcene	PPARA/CNR2
5	Terpinolene	PPARA/CNR2
6	2,3-Dehydro-1,8-cineole	HIF1A/NPC1L1/CYP19A1/TBXA2R/TTL/AR/NR1H3/NR1H2
7	Limonene	PPARA/CNR2/NR1H3/CYP19A1
8	Eucalyptol	CYP19A1/SHH/CYP51A1/ACHE
9	γ-Terpinene	TRPV1/PLK1/GLI2/GLI1
10	p-Cymene	CYP2A6/ACHE/TAAR1/PPARA/PTGS1/TRPA1
11	4-Carene	SLC6A2/SLC6A4/CYP2C19/PPARA/CNR2/ACHE/FAAH/TRPV1/AR/CYP19A1/ESR1/CHRM2
12	2-Nonanone	CYP19A1/CA2/CA1/CA4/CES1/CES2/AR/NR1I3/ADH1A/ADH1B/ADH7/SRD5A1/ADH1C/MAOA/MAOB/PGR/HLCS/ABCG2/DRD2/NOS2/MTNR1A/MTNR1B/SIRT2/PSMB5/EPHX2/CNR2/GSK3B/MMP13/MMP1/GRM5/NOTUM/CTSK/PTPRC/PTPRF/CDC25B/TRPA1/DBF4/ALOX5/CHRM1/CYP2C19/SLC6A2/SLC6A3/GSR/PAOX/IKBKB/CA7/JAK1/CA3/JAK2/CA12/CA14/CA9/CA13/CA5B/CHRM2/CHRM3/TTR/CYP17A1/PTGS1/SLC6A4/PTGS2/SRD5A2/ACHE/PARP1/NQO2/P2RX7/FABP4/FABP3/CDC7
13	Copaene	PPARA/CNR2/AR/CYP19A1/ESR1/CHRM2/ACHE/SLC6A2/SLC6A4/CYP2C19/BCHE/PTPN1/NR1I3/FAAH/TRPV1

14	4-Thujanol	TRPM8/CA2/CA1/CA4/AR/CDC25A/CDC25B/NR1I3/SHH/ESR1/ESR2/SHBG/UGT2B7/NR1H3/POLA1/NPC1L1/HSD11B1/NR3C1/NR1H4/GPBAR1/DRD2/NR3C2/PGR/SLC6A3/G6PD/GABBR1/IDO1
15	Pinocarvone	PTPN1/FAAH/TRPV1/ACHE/TRPA1
16	Borneol acetate	ACHE/VDR/PTPN1/HMGCR/CYP19A1/UGT2B7/CDC25A/TBXAS1/POLA1/CYP11B2/PABPC1/TYMS/CTRB1
17	Caryophyllene	PPARA/CNR2/FAAH/TRPV1
18	Terpinen-4-ol	AR/CYP19A1/CA2/CA1/CA4/RORC/SREBF2/NPC1L1/NR1H3/HMGCR/TRPM8/ESR2/CHRM2/CYP17A1/PTPN1/RORA/SQLE/DRD2/SLC6A4/CYP51A1/PPARA/PPARD/G6PD/ESR1/ACHE/ADRA2C/HSD17B2/SHBG/SLC6A2/NR3C1/NR3C2/BCHE/PTPRF/PTPN2/PLA2G1B/ACP1/AKR1B10
19	Dodecane, 2-methyl-	-
20	Myrtenal	CYP19A1/SRD5A2/FAAH/TRPV1/CYP17A1/ADH1A/SIGMAR1/AR/NPC1L1/PTGES/HSD17B3/ADORA3/MAPK3/PRKCH/HSD11B2/CDC25A/PTPN11/AKR1B10/PTGS1/BCHE/PTPN2/BACE1/CTSD/ESR1/ESR2/TRPA1/ADH1B/ADH1C/HSD11B1/RORA/PTPN6/FNTA/FNTB/FDFT1/DRD2/DRD4/PARP1/SRD5A1
21	Butanedioic acid, 2,3-bis(acetyloxy)-	KDM5A/JMJD1C/CA2/CA12/ABCB1
22	Cyclohexanol, 4-(1-methylethyl)-	CA2/CA1/CA4/TRPM8/AR/NR1I3/GPBAR1/SHH/UGT2B7/SHBG/ESR1/ESR2/CDC25A/NPC1L1/CDC25B/HSD11B1/SIGMAR1/DRD2/NR1H4/NR1H3/NR3C1/NR3C2/PGR/G6PD/GABBR1
23	(1S,3R,5S)-6,6-dimethyl-2-methylidenebicyclo[3.1.1]heptan-3-ol	UGT2B7/CA2/CA1/CA4/NR1H3/PTGS1/TRPM8/DRD2/HSD11B1/PTPN1/NR1I3
24	4-(1-methylethyl)-2-Cyclohexen-1-one	NR3C2/NR3C1/PGR/SERPINA6/SHBG/NR1I2/CYP17A1/HSD17B3/FABP1/NR1I3/SRD5A1/SRD5A2/SIGMAR1/CYP19A1/ADH1A/AR/FAAH/TRPV1/NPC1L1/PTGES/CCR5/ADORA3/MAPK3/PRKCH/HSD11B2/CDC25A/PTPN11/AKR1B10/PTGS1/BCHE/PTPN2/BACE1/CTSD/ESR1/ESR2/SLC6A3/TRPA1/ADH1B/ADH1C/RORA/FNTA/CES1/CHRNA4/CHRNB2/FNTB
25	Cyclohexanemethanol, α, α- dimethyl-4-methylene-	CA2/CA1/CA4/PTGS1/TRPM8/HSD11B1/UGT2B7/NR1H3/DRD2/AR/NR1I3/SAE1/AKR1B10/CYP19A1/TRPV3/HMOX1/HSD17B2/SLC6A4/ADRA2C/UBA2

26	Verbenol	AR/CA2/CA1/CA4/RORC/NPC1L1/RORA/TRPM8/SQLE/SHBG/NR1H3/PTPN1/POLB/NR1I3/SREBF2/CYP19A1/DRD2/BCHE/CHRM2/SLC6A4/NR3C1/ACHE/SRD5A2/PPARG/PPARA/PPARD/LTB4R
27	4a,8-Dimethyl-2-(prop-1-en-2-yl)-1,2,3,4,4a,5,6,7-octahydronaphthalene	PPARA/CNR2/NR1H3/AR/CYP19A1/ESR1/CHRM2/ACHE/SLC6A2/SLC6A4/CYP2C19/BCHE/ESR2/PTPN1/NR1I3
28	α-Terpineol	AR/CYP19A1/CA2/CA1/CA4/TRPM8/CHRM2/SLC6A4/NR1H3/PTPN1/NR1I3/SREBF2/NPC1L1/BCHE/ESR1/SQLE/ACHE/CYP51A1/SLC6A2/DRD2/CYP17A1/ESR2/CYP2C19/R3C2/PTPRF/PTPN2/PLA2G1B/ACP1/AKR1B10/SIGMAR1/NR3C1/TRPV3/ATP12A/PTPN6/SHBG/FABP4/PPARA/FABP3/FABP5/PPARD/FABP1/RORA/HMOX1/HMGCR/CD81/PGR/G6PD/SCD/ADRA2C/HSD11B1/SLC6A3
29	(3*R*,5a*R*,9*S*,9a*S*)-2,2,5a,9-Tetramethyloctahydro-2*H*-3,9a-methanobenzo[b]oxepine	CYP19A1/SHH/CYP51A1/ACHE
30	1-Cyclohexene-1-carboxaldehyde, 4-(1-methylethyl)-	SRD5A1/SERPINA6/SHBG/NR1I2/HSD17B3/FABP1/SRD5A2/CYP19A1/TRPV1/TRPA1/SIGMAR1/CCR5/ADH1A/PGR/AR/NR1I3/NPC1L1/PTGES/ADH4/ADH1B/ADH1C/NR3C2/CYP17A1/ADORA3/MAPK3/PRKCH/HSD11B2/CDC25A/PTPN11/AKR1B10/NR3C1/BCHE/BACE1/CTSD/SLC6A3/PTPN6/FAAH/RORA/FNTA/CNR1/PPARA/TERT/FABP5/PPARD/FDFT1/SLC5A7/ALDH1A1/CES1/PTPN1/FNTB
31	β-cadinene	PPARA/CNR2/NR1H3/CYP19A1
32	Cuminaldehyde	ADH1A/ADH4/ADH1B/ADH1C/TYR/CES1/CES2/ALDH1A1/GSK3B/SRD5A1
33	Myrtenol	SHBG/TRPM8/CYP19A1/SQLE/CHRM2/NR1H3/POLB/DRD2/SREBF2/SLC6A4/LTB4R/PTPN6/PTPN2/SPHK2/SPHK1/AR/CA2/CA1/CA4/RORC/NPC1L1/RORA/PTPN1/NR1I3
34	2*H*-3,9a-Methano-1-benzoxepin, octahydro-2,2,5a,9-tetramethyl-	CYP19A1/SHH/CYP51A1/ACHE
35	2-Cyclohexen-1-ol, 2-methyl-5-(1-methylethenyl)-	AR/CA2/CA1/CA4/SQLE/NR1H3/DRD2/TRPM8/NPC1L1/RORA/CYP19A1/TRPV3/HMOX1/SHBG/ADRA2C/HSD17B2/CHRM2/NR3C1/PTAFR/SCN5A/SCN9A/TOP1
36	2-(4-Methylphenyl)propan-2-ol	TRPM8/HMOX1/TRPV3/NR3C2/NR3C1/PGR/SLC6A3/OPRK1/CA2/SRD5A2/PTAFR/KCNA5/JAK1/JAK2/HSD17B2/CA4/CYP51A1
37	4-Epicubebol	UGT2B7/HSD11B1/SHBG/NR1I3/AR/CDC25A/CDC25B/ESR1/POLA1/GPBAR1/SHH/ESR2/NR1H3/TRPM8/CA2/CA

		1/NPC1L1/CA4/IDO1/CYP19A1/NR1H4/CYP17A1/SERPINA6/HSD17B3
38	α-Calacorene	NR1H4/NR1I2
39	Cubebol	UGT2B7/HSD11B1/AR/SHBG/NR1I3/POLA1/SHH/ESR1/ESR2/CDC25A/CDC25B/TRPM8/NR1H3/CA2/CA1/CA4/NPC1L1/GPBAR1/NR1H4/CYP19A1/IDO1/CYP17A1
40	Caryophyllene oxide	SQLE/LSS/SLC6A4/SLC6A3
41	1,3-Cyclohexadiene-1-methanol, 4-(1-methylethyl)-	PPARG/VDR/RBP4/CYP11B1/CYP11B2/JAK1/JAK2/GLRA1
42	1,5,5,8-Tetramethyl- 12- oxabicyclo[9.1.0]dodeca-3,7-diene	SQLE/AR/LSS/NR1H3/NR1H2/POLB/NPC1L1/KCNH2/NLRP3/POLA1/NR3C2/NR3C1/PGR/SERPINA6/SHBG/NR1I2/FABP1/NR1I3/SRD5A1/CYP19A1/SRD5A2/FAAH/TRPV1/CYP17A1/ADH1A/SIGMAR1/PTGES/HSD17B3/ADORA3/MAPK3/PRKCH/HSD11B2/CDC25A/PTPN11/AKR1B10/PTGS1/BCHE/PTPN2/BACE1/CTSD/ESR1/ESR2/TRPA1/ADH1B/ADH1C/HSD11B1/RORA/PTPN6/FNTA/FDFT1/DRD2/DRD4/PARP1/FNTB
43	1,4-Cyclohexadiene-1-methanol, 4-(1-methylethyl)-	SIGMAR1/DRD2/CYP11B1/PTGS2/CYP11B2/SQLE
44	Cyclohexanemethanol, 4-ethenyl-α,α,4-trimethyl-3- (1-methylethenyl)-	UGT2B7/HSD11B1/PTGS1/NR1H3/CYP19A1/SLC6A4/AR/CYP17A1/NR3C2/BCHE/SLC10A2/SAE1/AKR1B10/NR1I3/GCGR/ICMT/PSEN2/CNR2/PER2/SLC18A3/HSD17B2/HMGCR/PABPC1/ABHD6/UBA2/PSENEN/NCSTN/PSEN1/APH1B/APH1A
45	Guaiol	NR1I3/CYP19A1/PTPN1/SLC6A4/AR/ESR1/CHRM2/ACHE/SLC6A2/CYP2C19/NR1H3/SQLE/CYP17A1/CES2/BCHE/UGT2B7/HSD11B1/SREBF2/NPC1L1/FABP4/PPARA/TERT/FABP3/FABP5/PPARD/FABP1/CYP51A1/SHBG/HMGCR/POLB/PDE4D/PTPRF/PTPN2/PLA2G1B/ACP1/SCD/AKR1B10/CDC25B
46	Rosifoliol	HMGCR/SHBG/ESR2/SLC6A4/CHRM2/ACHE/SLC6A2/CYP2C19/NR1I3/RORA/BCHE/UGT2B7/G6PD/PTPN1/SERPINA6/PTPN6/PTPN2/SQLE/HSD11B1/CES2/PREP/IDO1/PTGER2/TBXAS1/FABP4/PPARG/PPARA/TERT/FABP3/FABP5/PPARD/FABP1/NR3C1/PABPC1/POLB/PDE4D/PTPRF/PLA2G1B/ACP1/AKR1B10/CYP19A1/NR1H3/CYP17A1/RORC/SREBF2/NPC1L1/CYP51A1/AR/ESR1
47	2-Naphthalenemethanol, 1,2,3,4,4a,5,6,7-octahydro- α,α, 4a,8-tetramethyl-	CYP19A1/PTPN1/AR/ESR1/CHRM2/ACHE/SLC6A2/SLC6A4/CYP2C19/NR1I3/NR1H3/CYP17A1/RORC/SREBF2/NPC1L1/CYP51A1/HSD11B1/HMGCR/POLB/PDE4D/PTPRF/PTPN2/PLA2G1B/ACP1/AKR1B10/BCHE/UGT2B7/CDC25

		B/PTPN6/SQLE/SHBG/ESR2/CD81/CES2/PREP/ATP12A/FNTA/FABP4/PPARG/PPARA/TERT/FABP3/FABP5/PPARD/FABP1/RORA/HSD11B2/G6PD/SCD/LTB4R/PTGS2/FNTB
48	1*H*-Cycloprop[e]azulen-7-ol, decahydro-1, 1,7-trimethyl- 4-methylene-, [1ar-(1aα,4aα,7β,7aβ,7bα)]-	UGT2B7/HSD11B1/IDO1/PTGS1/NR1H3/PGR/SLC6A3/ICMT
49	4a,8-Dimethyl-4,4a,5,6,7,8-hexahydronaphthalen-2(3*H*)-one	SRD5A1/SRD5A2/NR3C2/NR3C1/PGR/SERPINA6/SHBG/NR1I2/HSD17B3/FABP1/NR1I3/SIGMAR1/CYP19A1/AR/CYP17A1/NPC1L1/PTGES/TRPA1/CCR5/ADORA3/MAPK3/PRKCH/HSD11B2/CDC25A/PTPN11/AKR1B10/PTGS1/BCHE/PTPN2/BACE1/FAAH/TRPV1/CTSD/ESR1/ESR2/HSD11B1/SLC6A3/RORA/ADH1A/FNTA/FNTB/CES1/NOS2/ALOX5/CES2/PTPN6/PTPN1/FDFT1/ADH1C/TYMS/DRD4
50	1,7-di-iso-propylnaphthalene	CYP2A6/MGLL/CXCR3/TAAR1
51	1,3-di-iso-propylnaphthalene	HTR2B/GABRB3/GABRA1/GABRB2/GABRG2/SLC6A2
52	5-Azulenemethanol, 1,2,3,3a,4,5,6,7-octahydro-α,α, 3,8-tetramethyl-	CYP19A1/AR/ESR1/CHRM2/ACHE/SLC6A2/SLC6A4/CYP2C19/CYP17A1/NR1H3/NR1I3/RORC/SREBF2/NPC1L1/CYP51A1/HMGCR/SHBG/SQLE/BCHE/UGT2B7/ESR2/PTPN1/FABP4/PPARG/PPARA/TERT/FABP3/FABP5/PPARD/FABP1/RORA/HSD11B1/SCD/G6PD/SERPINA6/CES2/TOP1/PTGER2/ATP12A/PTPN6/PTPN2/CNR1/IDO1/GCGR/GRM5
53	Agarospirol	CYP19A1/SLC6A4/AR/ESR1/CHRM2/ACHE/SLC6A2/CYP2C19/CYP17A1/NR1H3/SREBF2/NPC1L1/CYP51A1/BCHE/HMGCR/SHBG/ESR2/NR1I3/SQLE/UGT2B7/RORC/PTPN1/PTGES/RORA/G6PD/CES2/PTPN6/PTPN2/SERPINA6/FABP4/PPARG/PPARA/TERT/FABP3/FABP5/PPARD/FABP1/SCD/HSD11B1/PREP/POLB/TOP1/PTGER2/ATP12A/NR3C1/DHCR7/SLC6A3/ICMT/PTPN11/AKR1B10
54	1,4-di-iso-propylnaphthalene	CYP2A6/TAAR1
55	*n*-Decanoic acid	FABP4/FABP5/FABP3/FFAR1/SLC22A6/HSD11B1/PPARA/AKR1B10/VDR/NR1H4/POLB/GPBAR1/AR/FABP2/PHF8/CYP19A1/PPARD/SERPINA6/SHBG/HSD17B3/G6PD/GABBR1/PTGER2/NPC1L1/CDC25A/CXCL8/HSD11B2/UGT2B7/CA2/CA1/KDM2A/KDM5C/HAO1/PLG/GSTK1/SRD5A1/SLC22A12/PTGDR2/CDC45/DUSP3/PTGDR/PTGFR/AKR1B1/TTR/AKR1C3/TBXA2R/GABRA2/PTPN1/BCAT2/FNTA/CACNA2D1/PTGS2/PDPK1/CHRNA7/RARA/IMPDH2/THRA/THRB/FABP1/GRM5/PTGS1/GABRB2/GABRG2/FNTB

56	2,4-Di-tert-butylphenol	CA2/PTGS1/HTR2B/HTR2C/GABRA1/SLC6A2/GABRB3/GABBR2/TNNC1/ESR2/HTR6/ESRRG/RORC/ESR1/AR/TRPA1/MC4R/TYR/ALOX5/AKT1/NOS1/NOS3/GABRG2/GABRB2/GABBR1/TNNT2/TNNI3
57	Dodecanoic acid	FFAR1/PPARA/FABP4/FABP5/PPARD/FABP3/HSD11B1/SLC22A6/AKR1B10/FABP2/VDR/AR/PTGER2/UGT2B7/PHF8/SERPINA6/SHBG/HSD17B3/G6PD/GABBR1/CDC25A/NR1H4/CYP19A1/NPC1L1/GPBAR1/KDM5C/FNTA/KDM2A/PTPN1/POLB/PTGFR/CA2/CA1/HAO1/GSTK1/LTA4H/HSD11B2/FFAR4/PLG/PTGDR2/GABRA2/CDC45/PTGER4/PTGER3/AKR1B1/CYP1A2/MAPK1/TTR/TBXA2R/RXRA/HMGCR/IMPDH2/SLC22A12/CA12/PTPRC/CACNA2D1/RBP4/EDNRA/RARA/THRA/THRB/PTGDR/SLC16A1/AKR1C3/CHRNA7/TRPA1/RARG/RARB/CXCL8/SLC6A4/MMP13/MMP9/MMP2/MMP14/MMP12/PTGES/CMA1/CTSG/AKR1A1/SAE1/GRM5/FABP1/STS/GSR/BMP1/GABRB2/GABRG2/FNTB/UBA2

Table S2. Disease-target gene database

NO.	Gene	NO.	Gene	NO.	Gene	NO.	Gene	NO.	Gene	NO.	Gene	NO.	Gene	NO.	Gene	NO.	Gene	NO.	Gene
1	ABCA1	2	ABCA3	3	ABCA4	4	ABCA7	5	ABCB1	6	ABCB11	7	ABCB4	8	ABCC6	9	ABCC8	10	ABCC9
11	ABCD1	12	ABCG5	13	ABCG8	14	ABHD5	15	ABL1	16	ABO	17	ACE	18	ACE2	19	ACHE	20	ACTA1
21	ACTA2	22	ACTC1	23	ACTN2	24	ACVRL1	25	ADA	26	ADA2	27	ADAM10	28	ADAM17	29	ADAMTS13	30	ADAMTS7
31	ADD1	32	ADH1C	33	ADIPOQ	34	ADM	35	ADORA2A	36	ADRA2C	37	ADRB1	38	ADRB2	39	ADTRP	40	AFG3L2
41	AGER	42	AGL	43	AGT	44	AGTR1	45	AGTR2	46	AIFM1	47	AKAP13	48	AKT1	49	ALB	50	ALDH1A2
51	ALDH2	52	ALMS1	53	ALOX5	54	ALOX5AP	55	AMPD1	56	ANGPT1	57	ANGPT2	58	ANGPTL4	59	ANK2	60	ANKRD1
61	ANKS1A	62	ANO5	63	ANXA5	64	APBB1	65	APC	66	APCS	67	APLN	68	APOA1	69	APOA1-AS	70	APOA2
71	APOA4	72	APOA5	73	APOB	74	APOC1	75	APOC3	76	APOE	77	APOH	78	APOL1	79	APP	80	AR
81	ARHGEF26	82	ARL15	83	ARNTL	84	ARPC1B	85	ARSA	86	ARSB	87	AS3MT	88	ASAH1	89	ASPA	90	ATG16L1
91	ATM	92	ATP1A1	93	ATP2A2	94	ATP2B1	95	ATP2C1	96	ATP7A	97	ATXN2	98	ATXN3	99	AVP	100	B2M
101	BAG3	102	BAZ1B	103	BBS1	104	BBS2	105	BCHE	106	BCKDHB	107	BCL2	108	BCS1L	109	BDNF	110	BGLAP
111	BGN	112	BLK	113	BMP2	114	BMP4	115	BMP6	116	BMPR1A	117	BMPR2	118	BRAF	119	BRAP	120	BRCA1
121	BRCA2	122	BRF1	123	BSCL2	124	BSND	125	BTNL2	126	C3	127	C4A	128	C9orf72	129	CACNA1A	130	CACNA1C
131	CACNB2	132	CADM3	133	CALCA	134	CALR	135	CARD9	136	CARMAL	137	CASP1	138	CASP3	139	CASP8	140	CASQ2
141	CASR	142	CAT	143	CAV1	144	CAV3	145	CBL	146	CBS	147	CCDC65	148	CCDC92	149	CCL11	150	CCL2
151	CCL3	152	CCL5	153	CCN2	154	CCND1	155	CCR1	156	CCR5	157	CD14	158	CD28	159	CD34	160	CD36
161	CD4	162	CD40	163	CD40LG	164	CD8A	165	CDH1	166	CDH13	167	CDH2	168	CDH23	169	CDK13	170	CDK8
171	CDKN1A	172	CDKN1C	173	CDKN2A	174	CDKN2B	175	CDKN2B-AS1	176	CELA2A	177	CELSR2	178	CEP290	179	CERS1	180	CETP
181	CFC1	182	CFDP1	183	CFH	184	CFTR	185	CHAT	186	CHCHD2	187	CHD7	188	CHDS1	189	CHDS2	190	CHDS3

191	CHDS4	192	CHDS8	193	CHDS9	194	CHGA	195	CIITA	196	CITED2	197	CKM	198	CLCN1	199	CLCN5	200	CLN3
201	CLU	202	CMA1	203	CNNM2	204	COG2	205	COL17A1	206	COL1A1	207	COL1A2	208	COL2A1	209	COL3A1	210	COL4A1
211	COL4A2	212	COL4A3	213	COL4A4	214	COL7A1	215	COMT	216	COPA	217	CORIN	218	COX10	219	CP	220	CPT2
221	CRB2	222	CRELD1	223	CRP	224	CRYAA	225	CRYAB	226	CSF2	227	CSF3	228	CSRP3	229	CST3	230	CTLA4
231	CTNNB1	232	CTSD	233	CX3CR1	234	CXCL12	235	CXCL8	236	CXCR4	237	CYBA	238	CYBB	239	CYCS	240	CYP11B2
241	CYP17A1	242	CYP27A1	243	CYP2C19	244	CYP2C9	245	CYP2D6	246	CYP3A4	247	CYP7A1	248	DAB2IP	249	DBT	250	DDAH2
251	DES	252	DHX16	253	DMD	254	DNAH11	255	DNAH5	256	DNAI1	257	DNAJB11	258	DNAJC6	259	DNM2	260	DNMT3A
261	DOCK6	262	DRD2	263	DSC2	264	DSG2	265	DSP	266	DTNA	267	DYNC2H1	268	DYNC2LI1	269	DYRK1A	270	DZIP1L
271	ECE1	272	ECM1	273	EDN1	274	EDN3	275	EDNRA	276	EDNRB	277	EGF	278	EGFR	279	EIF4G1	280	ELANE
281	ELN	282	ELOVL4	283	EMD	284	ENG	285	ENO2	286	ENPP1	287	EP300	288	EPHB4	289	EPHX2	290	EPO
291	ERBB2	292	ERBB3	293	ERBB4	294	ERCC2	295	ERCC6	296	ESR1	297	ESR2	298	EVC2	299	EYA4	300	F10
301	F11	302	F12	303	F13A1	304	F2	305	F3	306	F5	307	F7	308	F8	309	F9	310	FABP3
311	FADS1	312	FADS2	313	FARSB	314	FAS	315	FASLG	316	FASTKD2	317	FBLN5	318	FBN1	319	FBN2	320	FCGR2A
321	FCGR3B	322	FGA	323	FGB	324	FGF2	325	FGF23	326	FGFR1	327	FGFR2	328	FHL1	329	FIG4	330	FKRP
331	FKTN	332	FLNA	333	FLNC	334	FLT1	335	FLT4	336	FN1	337	FOS	338	FOXC1	339	FOXC2	340	FOXD3
341	FOXE3	342	FOXP1	343	FOXP3	344	FTL	345	FTO	346	FURIN	347	FUS	348	FXN	349	G6PC1	350	G6PD
351	GAA	352	GALNS	353	GALNT2	354	GAPDH	355	GARS1	356	GATA1	357	GATA3	358	GATA4	359	GATA5	360	GATA6
361	GATAD1	362	GBA	363	GBA1	364	GBE1	365	GCG	366	GCK	367	GCKR	368	GCLC	369	GDAP1	370	GDF1
371	GDF15	372	GDNF	373	GFAP	374	GGT1	375	GHR	376	GHRL	377	GIGYF2	378	GIP	379	GJA1	380	GJA5
381	GLA	382	GLB1	383	GLE1	384	GNAS	385	GNB3	386	GNB4	387	GNE	388	GNPTAB	389	GP1BA	390	GP6
391	GPT	392	GRK2	393	GRN	394	GSN	395	GSR	396	GSTM1	397	GTF2IRD1	398	GUCY1A1	399	GUSB	400	GYG1

401	GYS1	402	H19	403	H2AC18	404	HADHA	405	HAMP	406	HAND1	407	HAND2	408	HARS1	409	HBA1	410	HBA2
411	HBB	412	HCN4	413	HDAC9	414	HECTD4	415	HEG1	416	HEXB	417	HEY2	418	HFE	419	HGF	420	HGSNAT
421	HHIPL1	422	HIF1A	423	HJV	424	HLA-A	425	HLA-B	426	HLA-C	427	HLA-DPB1	428	HLA-DQA1	429	HLA-DQB1	430	HLA-DRB1
431	HMGB1	432	HMGCR	433	HMOX1	434	HNF1A	435	HNF1B	436	HNRNPA1	437	HNRNPA2B1	438	HNRNPUL2-BSCL2	439	HOXD10	440	HP
441	HRAS	442	HSD17B10	443	HSPA4	444	HSPB1	445	HSPB8	446	HSPD1	447	HSPG2	448	HTR2A	449	HTRA2	450	HTT
451	ICAM1	452	IDS	453	IDUA	454	IFIH1	455	IFNA1	456	IFNG	457	IFNGR1	458	IFT140	459	IFT172	460	IGF1
461	IGF1R	462	IGF2	463	IGF2R	464	IGFBP3	465	IKBKG	466	IL10	467	IL12A	468	IL12B	469	IL12RB1	470	IL13
471	IL17A	472	IL18	473	IL1A	474	IL1B	475	IL1R1	476	IL1RN	477	IL2	478	IL23R	479	IL2RA	480	IL37
481	IL4	482	IL5	483	IL6	484	IL6R	485	INAVA	486	INF2	487	INS	488	INSR	489	INVS	490	IQCB1
491	IRAK1	492	IRF5	493	IRGM	494	IRS1	495	ISL1	496	ITGA1	497	ITGA2	498	ITGA2B	499	ITGA3	500	ITGAM
501	ITGB2	502	ITGB3	503	JAG1	504	JAK2	505	JCAD	506	JPH1	507	JUN	508	JUP	509	KALRN	510	KAT6B
511	KCNA5	512	KCND3	513	KCNE1	514	KCNE2	515	KCNH2	516	KCNJ11	517	KCNJ13	518	KCNJ2	519	KCNJ5	520	KCNJ8
521	KCNK5	522	KCNQ1	523	KCTD10	524	KDR	525	KIF1B	526	KIT	527	KL	528	KNG1	529	KRAS	530	KRT5
531	LAMA2	532	LAMP2	533	LBR	534	LCAT	535	LCN2	536	LDB3	537	LDLR	538	LDLRAP1	539	LDLR-AS1	540	LEP
541	LEPR	542	LGALS2	543	LGALS3	544	LIPA	545	LIPC	546	LITAF	547	LMNA	548	LMOD1	549	LMX1B	550	LOC106627981
551	LOX	552	LPA	553	LPAL2	554	LPL	555	LRP1	556	LRP2	557	LRP5	558	LRP6	559	LRP8	560	LRRC56
561	LRSAM1	562	LTA	563	LTBP2	564	LZTR1	565	MACO1	566	MAP2K1	567	MAP2K2	568	MAP3K7CL	569	MAPK1	570	MAPK14
571	MAPT	572	MARS1	573	MB	574	MBL2	575	MC4R	576	MECP2	577	MED12	578	MED13L	579	MED25	580	MEF2A
581	MEF2C	582	MEFV	583	MEN1	584	MFN2	585	MGP	586	MHRT	587	MIA3	588	MIAT	589	MIF	590	MIR106B
591	MIR122	592	MIR126	593	MIR132	594	MIR140	595	MIR142	596	MIR143	597	MIR144	598	MIR145	599	MIR146A	600	MIR146B

601	MIR150	602	MIR155	603	MIR15A	604	MIR17	605	MIR17HG	606	MIR182	607	MIR196A2	608	MIR199A1	609	MIR208A	610	MIR21
611	MIR210	612	MIR22	613	MIR221	614	MIR222	615	MIR223	616	MIR29A	617	MIR29B1	618	MIR320A	619	MIR328	620	MIR34A
621	MIR423	622	MIR499A	623	MIR92B	624	MKKS	625	MLXIPL	626	MMACHC	627	MME	628	MMP1	629	MMP12	630	MMP13
631	MMP2	632	MMP3	633	MMP8	634	MMP9	635	MORC2	636	MPL	637	MPO	638	MPV17	639	MPZ	640	MRAS
641	MRPS6	642	MRTFA	643	MT-ATP6	644	MT-CO1	645	MT-CO2	646	MT-CO3	647	MT-CYB	648	MTHFD1L	649	MTHFR	650	MT-ND1
651	MT-ND2	652	MT-ND3	653	MT-ND4	654	MT-ND5	655	MT-ND6	656	MTOR	657	MTR	658	MTRR	659	MT-TK	660	MT-TL1
661	MTTP	662	MT-TS1	663	MUC1	664	MYBPC3	665	MYC	666	MYD88	667	MYH11	668	MYH6	669	MYH7	670	MYH9
671	MYL2	672	MYL3	673	MYLK	674	MYOCD	675	MYOT	676	MYPN	677	MYRF	678	NAGA	679	NAGLU	680	NAMPT
681	NAT2	682	NCF1	683	NCF2	684	NCF4	685	NDP	686	NDRG1	687	NDUFAF6	688	NDUFS4	689	NDUFV1	690	NEB
691	NEFL	692	NEU1	693	NEXN	694	NF1	695	NFE2L2	696	NFKB1	697	NGF	698	NHLRC1	699	NIPBL	700	NIPSNAP3B
701	NKX2-5	702	NKX2-6	703	NLRP3	704	NOD2	705	NODAL	706	NOS1	707	NOS2	708	NOS3	709	NOTCH1	710	NOTCH2
711	NOTCH2NLC	712	NOTCH3	713	NPC1	714	NPC1L1	715	NPC2	716	NPPA	717	NPPB	718	NPPC	719	NPY	720	NR1H3
721	NR1H4	722	NR2F2	723	NR3C1	724	NR3C2	725	NR4A2	726	NRAS	727	NRG1	728	NSD1	729	NTRK1	730	OFD1
731	OLR1	732	OPA1	733	OPTN	734	OXTR	735	P2RY12	736	PAH	737	PAPPA	738	PARK7	739	PAX2	740	PAX6
741	PCSK9	742	PDCD1	743	PDE11A	744	PDE5A	745	PDE8B	746	PDGFB	747	PDGFD	748	PDGFRA	749	PDGFRB	750	PECAM1
751	PEX1	752	PEX2	753	PEX5	754	PEX6	755	PEX7	756	PF4	757	PGAM2	758	PGF	759	PHACTR1	760	PHKA2
761	PHKG2	762	PHOX2B	763	PHYH	764	PIGL	765	PIK3C2A	766	PIK3CA	767	PINK1	768	PITX2	769	PKD1	770	PKD1L1
771	PKD2	772	PKHD1	773	PKP2	774	PLA2G2A	775	PLA2G6	776	PLA2G7	777	PLAT	778	PLAU	779	PLEC	780	PLEKHG5
781	PLG	782	PLN	783	PLOD1	784	PLPP3	785	PLTP	786	PLXND1	787	PMM2	788	PMP2	789	PMS2	790	PNPLA2
791	POGLUT1	792	POLG	793	POMC	794	POMGNT1	795	PON1	796	PON2	797	PON3	798	PPARA	799	PPARG	800	PPARGC1A
801	PPBP	802	PPM1K	803	PPP1CB	804	PRDM16	805	PRF1	806	PRKACA	807	PRKAG2	808	PRKAR1A	809	PRKCE	810	PRKCQ

811	PRKCSH	812	PRKG1	813	PRKN	814	PROCR	815	PRODH	816	PROM1	817	PRPS1	818	PSAP	819	PSEN1	820	PSEN2
821	PSMA6	822	PTCH1	823	PTEN	824	PTGS1	825	PTGS2	826	PTH	827	PTPN11	828	PTPN22	829	PTPRC	830	PTX3
831	PYGL	832	PYGM	833	RAB7A	834	RAF1	835	RBCK1	836	RBM20	837	RBP4	838	REN	839	RERE	840	RETN
841	RHCE	842	RHD	843	RHO	844	RHOA	845	RIPK1	846	RIT1	847	RNF213	848	RPGR	849	RPGRIP1L	850	RPL36A-HNRNPH2
851	RRM2B	852	RUNX1	853	RUNX2	854	RYR1	855	RYR2	856	S100B	857	SAA1	858	SAG	859	SAR1B	860	SBF2
861	SCARB1	862	SCN10A	863	SCN5A	864	SCNN1A	865	SCO2	866	SDHA	867	SDHB	868	SDHD	869	SEC61A1	870	SEC63
871	SELE	872	SELENON	873	SELL	874	SELP	875	SERPINA1	876	SERPINA3	877	SERPINC1	878	SERPINE1	879	SERPINF1	880	SETBP1
881	SFRP4	882	SFTPC	883	SGCB	884	SGCD	885	SGSH	886	SH2B3	887	SH2D1A	888	SH3TC2	889	SHH	890	SHOC2
891	SLC11A1	892	SLC12A3	893	SLC17A5	894	SLC19A3	895	SLC22A3	896	SLC22A5	897	SLC25A4	898	SLC29A3	899	SLC2A1	900	SLC2A10
901	SLC34A1	902	SLC37A4	903	SLC40A1	904	SLC6A2	905	SLC6A4	906	SLC8A1	907	SLURP1	908	SMAD2	909	SMAD3	910	SMAD4
911	SMAD6	912	SMARCA4	913	SMC1A	914	SMPD1	915	SNCA	916	SOD1	917	SOD2	918	SOD3	919	SORL1	920	SORT1
921	SOS1	922	SOX9	923	SP110	924	SPP1	925	SQSTM1	926	SREBF1	927	SST	928	STAT1	929	STAT3	930	STAT4
931	STN1	932	STX1A	933	SURF1	934	SVEP1	935	TAB2	936	TAFAZZIN	937	TARDBP	938	TBK1	939	TBL2	940	TBP
941	TBX1	942	TBX18	943	TBX2	944	TBX20	945	TBX4	946	TBX5	947	TCAP	948	TCF21	949	TCF7L2	950	TEK
951	TERC	952	TERT	953	TET2	954	TF	955	TG	956	TGFB1	957	TGFB2	958	TGFB3	959	TGFBR1	960	TGFBR2
961	TH	962	THBD	963	THBS1	964	THSD7A	965	TIMP1	966	TKT	967	TLL1	968	TLR2	969	TLR4	970	TLR5
971	TMEM216	972	TMEM43	973	TMEM67	974	TNF	975	TNFAIP3	976	TNFRSF11A	977	TNFRSF11B	978	TNFRSF1A	979	TNFRSF1B	980	TNFSF11
981	TNFSF4	982	TNNC1	983	TNNI3	984	TNNI3K	985	TNNT2	986	TOMM40	987	TP53	988	TPM1	989	TRAF6	990	TRDN
991	TRIB1	992	TRIM2	993	TRPM4	994	TRPV4	995	TSC1	996	TSC2	997	TSFM	998	TSHR	999	TTN	1000	TTN-AS1
1001	TTR	1002	TWNK	1003	TYMP	1004	UBE2Z	1005	UCHL1	1006	UCP2	1007	UGT1A1	1008	UMOD	1009	UTS2	1010	VCAM1

1011	VCL	1012	VCP	1013	VDR	1014	VEGFA	1015	VHL	1016	VKORC1	1017	VPS13C	1018	VWF	1019	WDPCP	1020	WDR12
1021	WDR35	1022	WFS1	1023	WT1	1024	XDH	1025	XIAP	1026	XK	1027	XYLT1	1028	XYLT2	1029	YARS1	1030	YY1AP1
1031	ZAP70	1032	ZC3HC1	1033	ZEB2	1034	ZFAT	1035	ZFHX3	1036	ZFPM2	1037	ZIC3	1038	ZMPSTE24	1039	ZNF687	1040	ZPR1

Table S3. Target genes interacting with both chemicals and coronary heart disease

No.	Chemical constituents	Disease-related targets
1	*β*-Pinene	ESR1/AR/PPARA/CYP2C19/SLC6A4/PTGS1/SLC6A2/ACHE/NR1H3
2	Sabinene	ESR1/AR/PPARA/CYP2C19/SLC6A4/PTGS1/SLC6A2/NR1H4/ACHE/NR1H3
3	*α*-Phellandrene	PPARA/ADORA2A
4	*β*-Myrcene	PPARA
5	Terpinolene	PPARA
6	2,3-Dehydro-1,8-cineole	AR/HIF1A/NPC1L1/NR1H3
7	Limonene	PPARA/NR1H3
8	Eucalyptol	SHH/ACHE
9	*p*-Cymene	PPARA/PTGS1/ACHE
10	4-Carene	ESR1/AR/PPARA/CYP2C19/SLC6A4/SLC6A2/ACHE
11	2-Nonanone	TTR/MMP1/NOS2/JAK2/AR/FABP3/PTGS2/CYP2C19/SLC6A4/PTGS1/SLC6A2/ADH1C/EPHX2/ACHE/GSR/PTPRC/ALOX5/MMP13/DRD2/CYP17A1
12	Copaene	ESR1/AR/PPARA/CYP2C19/SLC6A4/SLC6A2/BCHE/ACHE
13	4-Thujanol	ESR1/AR/NR3C2/G6PD/SHH/NR1H4/NR3C1/NPC1L1/ESR2/DRD2/NR1H3
14	Pinocarvone	ACHE
15	Borneol acetate	HMGCR/VDR/CYP11B2/ACHE

16	Caryophyllene	PPARA
17	Terpinen-4-ol	ESR1/HMGCR/AR/PPARA/NR3C2/G6PD/SLC6A4/SLC6A2/BCHE/ACHE/NR3C1/NPC1L1/ESR2/DRD2/ADRA2C/NR1H3/CYP17A1
18	Myrtenal	ESR1/PTPN11/AR/CTSD/PTGS1/ADH1C/BCHE/NPC1L1/ESR2/DRD2/CYP17A1
19	Butanedioic acid, 2,3-bis(acetyloxy)-	ABCB1
20	Cyclohexanol, 4-(1-methylethyl)-	ESR1/AR/NR3C2/G6PD/SHH/NR1H4/NR3C1/NPC1L1/ESR2/DRD2/NR1H3
21	(1S,3R,5S)-6,6-dimethyl-2-methylidenebicyclo[3.1.1]heptan-3-ol	PTGS1/DRD2/NR1H3
22	4-(1-methylethyl)-2-Cyclohexen-1-one	ESR1/PTPN11/AR/CCR5/NR3C2/CTSD/PTGS1/ADH1C/BCHE/NR3C1/NPC1L1/ESR2/CYP17A1
23	Cyclohexanemethanol, α, α- dimethyl-4-methylene-	HMOX1/AR/SLC6A4/PTGS1/DRD2/ADRA2C/NR1H3
24	Verbenol	PPARG/AR/PPARA/SLC6A4/BCHE/ACHE/NR3C1/NPC1L1/DRD2/NR1H3
25	4a,8-Dimethyl-2-(prop-1-en-2-yl)-1,2,3,4,4a,5,6,7-octahydronaphthalene	ESR1/AR/PPARA/CYP2C19/SLC6A4/SLC6A2/BCHE/ACHE/ESR2/NR1H3
26	α-Terpineol	ESR1/HMOX1/HMGCR/AR/FABP3/PPARA/NR3C2/CYP2C19/G6PD/SLC6A4/SLC6A2/BCHE/ACHE/NR3C1/NPC1L1/ESR2/DRD2/ADRA2C/NR1H3/CYP17A1
27	(3*R*,5a*R*,9*S*,9a*S*)-2,2,5a,9-Tetramethyloctahydro-2*H*-3,9a-methanobenzo[b]oxepine	SHH/ACHE
28	1-Cyclohexene-1-carboxaldehyde, 4-(1-methylethyl)-	PTPN11/AR/TERT/PPARA/CCR5/NR3C2/CTSD/ADH1C/BCHE/NR3C1/NPC1L1/CYP17A1
29	Cuminaldehyde	ADH1C
30	Myrtenol	AR/SLC6A4/NPC1L1/DRD2/NR1H3

31	2*H*-3,9a-Methano-1-benzoxepin, octahydro-2,2,5a,9-tetramethyl-	SHH/ACHE
32	2-Cyclohexen-1-ol, 2-methyl-5-(1-methylethenyl)-	SCN5A/HMOX1/AR/NR3C1/NPC1L1/DRD2/ADRA2C/NR1H3
33	2-(4-Methylphenyl)propan-2-ol	HMOX1/JAK2/NR3C2/NR3C1/KCNA5
34	4-Epicubebol	ESR1/AR/SHH/NR1H4/NPC1L1/ESR2/NR1H3/CYP17A1/
35	α-Calacorene	NR1H4
36	Cubebol	ESR1/AR/SHH/NR1H4/NPC1L1/ESR2/NR1H3/CYP17A1
37	Caryophyllene oxide	SLC6A4
38	1,3-Cyclohexadiene-1-methanol, 4-(1-methylethyl)-	PPARG/JAK2/VDR/RBP4/CYP11B2
39	1,5,5,8-Tetramethyl- 12- oxabicyclo[9.1.0]dodeca-3,7-diene	ESR1/PTPN11/KCNH2/AR/NLRP3/NR3C2/CTSD/PTGS1/ADH1C/BCHE/NR3C1/NPC1L1/ESR2/DRD2/NR1H3/CYP17A1
40	1,4-Cyclohexadiene-1-methanol, 4-(1-methylethyl)-	PTGS2/CYP11B2/DRD2
41	Cyclohexanemethanol, 4-ethenyl-α,α,4-trimethyl-3- (1-methylethenyl)-	PSEN1/PSEN2/HMGCR/AR/NR3C2/SLC6A4/PTGS1/BCHE/NR1H3/CYP17A1
42	Guaiol	ESR1/HMGCR/AR/FABP3/TERT/PPARA/CYP2C19/SLC6A4/SLC6A2/BCHE/ACHE/NPC1L1/NR1H3/CYP17A1
43	Rosifoliol	PPARG/ESR1/HMGCR/AR/FABP3/TERT/PPARA/CYP2C19/G6PD/SLC6A4/SLC6A2/BCHE/ACHE/NR3C1/NPC1L1/ESR2/NR1H3/CYP17A1
44	2-Naphthalenemethanol, 1,2,3,4,4a,5,6,7-octahydro- α,α, 4a,8-tetramethyl-	PPARG/ESR1/HMGCR/AR/FABP3/TERT/PPARA/PTGS2/CYP2C19/G6PD/SLC6A4/SLC6A2/BCHE/ACHE/NPC1L1/ESR2/NR1H3/CYP17A1
45	1*H*-Cycloprop[e]azulen-7-ol, decahydro-1, 1,7-trimethyl- 4-methylene-, [1ar-	PTGS1/NR1H3

	(1aα,4aα,7β,7aβ,7bα)]-	
46	(4a*S*,8*R*)-4a,8-Dimethyl-4,4a,5,6,7,8-hexahydronaphthalen-2(3*H*)-one	ESR1/PTPN11/NOS2/AR/CCR5/NR3C2/CTSD/PTGS1/ADH1C/BCHE/NR3C1/NPC1L1/ESR2/ALOX5/CYP17A1
47	1,3-di-iso-propylnaphthalene	SLC6A2
48	5-Azulenemethanol, 1,2,3,3a,4,5,6,7-octahydro-α,α, 3,8-tetramethyl-	PPARG/ESR1/HMGCR/AR/FABP3/TERT/PPARA/CYP2C19/G6PD/SLC6A4/SLC6A2/BCHE/ACHE/NPC1L1/ESR2/NR1H3/CYP17A1
49	Agarospirol	PPARG/ESR1/PTPN11/HMGCR/AR/FABP3/TERT/PPARA/CYP2C19/G6PD/SLC6A4/SLC6A2/BCHE/ACHE/NR3C1/NPC1L1/ESR2/NR1H3/CYP17A1
50	*n*-Decanoic acid	TTR/CXCL8/AR/FABP3/PPARA/PTGS2/PLG/G6PD/VDR/PTGS1/NR1H4/NPC1L1
51	2,4-Di-tert-butylphenol	NOS3/TNNT2/TNNI3/ESR1/AKT1/AR/PTGS1/SLC6A2/NOS1/MC4R/ESR2/TNNC1/ALOX5
52	Dodecanoic acid	MAPK1/MMP9/TTR/CXCL8/MMP2/HMGCR/EDNRA/AR/FABP3/PPARA/PLG/G6PD/SLC6A4/VDR/RBP4/NR1H4/MMP12/GSR/NPC1L1/CMA1/PTPRC/MMP13

Table S4. The content of 10 ingredients in 20 batches of *Oleum Cinnamomi* (mg/g, *n*=3)

Batch	*β*-Pinene	Sabinene	Limonene	Eucalyptol	*p*-Cymene	Terpinen-4-ol	4-(1-methylethyl)-2-Cyclohexen-1-one	*α*-Terpineol	Caryophyllene oxide	Guaiol
S1	27.60	32.80	5.14	165.35	65.13	8.45	1.12	49.97	38.99	9.82
S2	33.79	43.47	6.36	189.73	73.22	7.33	1.02	51.08	36.38	9.38
S3	38.68	48.19	6.91	221.28	87.35	7.83	2.13	48.77	34.41	4.86
S4	45.66	58.12	7.21	207.34	88.71	7.87	0.91	49.01	31.60	8.43
S5	48.96	61.68	6.90	193.10	85.42	5.67	0.69	39.31	34.28	8.50
S6	47.66	57.34	6.31	201.58	80.66	6.13	0.69	40.90	32.89	8.27
S7	46.35	59.67	7.03	215.96	83.70	4.44	0.79	41.14	36.74	6.38
S8	43.20	55.25	6.20	198.06	84.90	5.30	1.21	36.60	35.70	5.07
S9	47.54	62.96	6.76	196.00	84.37	5.31	0.69	39.68	35.41	7.72
S10	10.28	3.04	2.98	65.69	15.29	6.17	4.72	21.21	11.51	1.75
S11	13.89	3.19	2.75	67.24	18.01	7.43	4.85	23.20	11.84	1.79
S12	20.21	14.13	6.67	103.04	17.69	7.87	3.81	28.08	16.83	3.49
S13	10.46	2.20	5.62	88.26	17.96	6.38	4.36	14.01	13.67	0.54
S14	10.22	2.51	8.75	112.70	18.06	7.80	4.22	16.91	12.75	0.49
S15	9.39	2.19	8.70	111.71	16.81	8.27	3.95	16.79	12.96	0.51
S16	8.89	3.73	4.07	63.90	16.15	7.58	3.14	17.73	20.72	5.16
S17	11.08	2.22	6.77	117.40	21.7	10.04	5.52	18.96	14.94	0.44
S18	8.69	1.81	7.80	108.67	16.54	8.19	4.72	18.11	14.87	0.54
S19	13.29	2.51	11.73	114.17	21.60	10.47	5.04	19.61	11.74	0.81
S20	8.93	1.90	6.85	104.13	17.01	7.96	4.95	16.78	14.92	0.49

Table S5. Sample information of *Fructus Cinnamomi*

Batch	Production Place	Batch	Production Place
S1	Luodian, Qiannan, Guizhou	S11	Libo, Qiannan, Guizhou
S2	Luodian, Qiannan, Guizhou	S12	Baoshan, Yunnan
S3	Luodian, Qiannan, Guizhou	S13	Pu'er, Yunnan
S4	Luodian, Qiannan, Guizhou	S14	Jiangcheng, Yunnan
S5	Luodian, Qiannan, Guizhou	S15	Mile, Yunnan
S6	Luodian, Qiannan, Guizhou	S16	Lingyun, Guangxi
S7	Luodian, Qiannan, Guizhou	S17	Leye, Guangxi
S8	Luodian, Qiannan, Guizhou	S18	Pingguo, Guangxi
S9	Luodian, Qiannan, Guizhou	S19	Tiandong, Guangxi
S10	Guanling, Anshun, Guizhou	S20	Jingxi, Guangxi

Table S6. Ion information of multi-index components in *Oleum Cinnamomi*

Chemical composition	Target ion (*m*/*z*)	Reference ion (*m*/*z*)	Reference ion (*m*/*z*)
β-pinene	79	91	41
Sabinene	93	91	77
Limonene	68	93	67
Eucalyptol	71	80	108
p-Cymene	117	134	91
Terpinen-4-ol	71	93	111
4-(1-methylethyl)-2-cyclohexen-1-one	96	95	67
α-Terpineol	121	93	59
Caryophyllene oxide	79	93	43
Guaiol	161	105	107
Naphthalene (IS[1])	128	127	102
Cyclohexanone (IS[2])	55	42	98

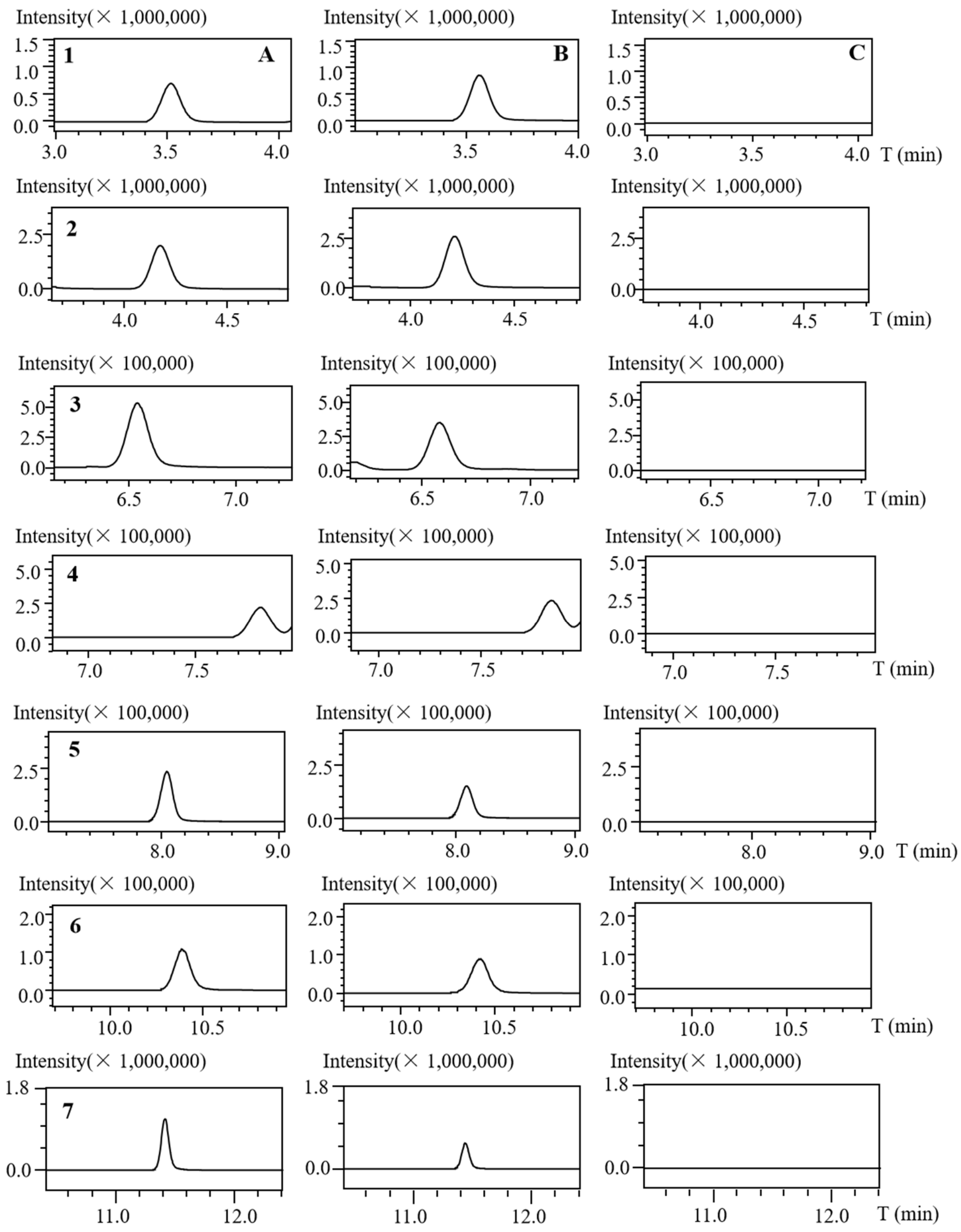

Figure S1. GC-MS SIM chromatogram of reference solution (A), sample solution (B) and blank control solution (C)

Note: 1: β-Pinene, 2: Sabinene, 3: β-Myrcene, 4: Limonene, 5: Eucalyptol, 6: γ-Terpinene, 7: *p*-Cymene.

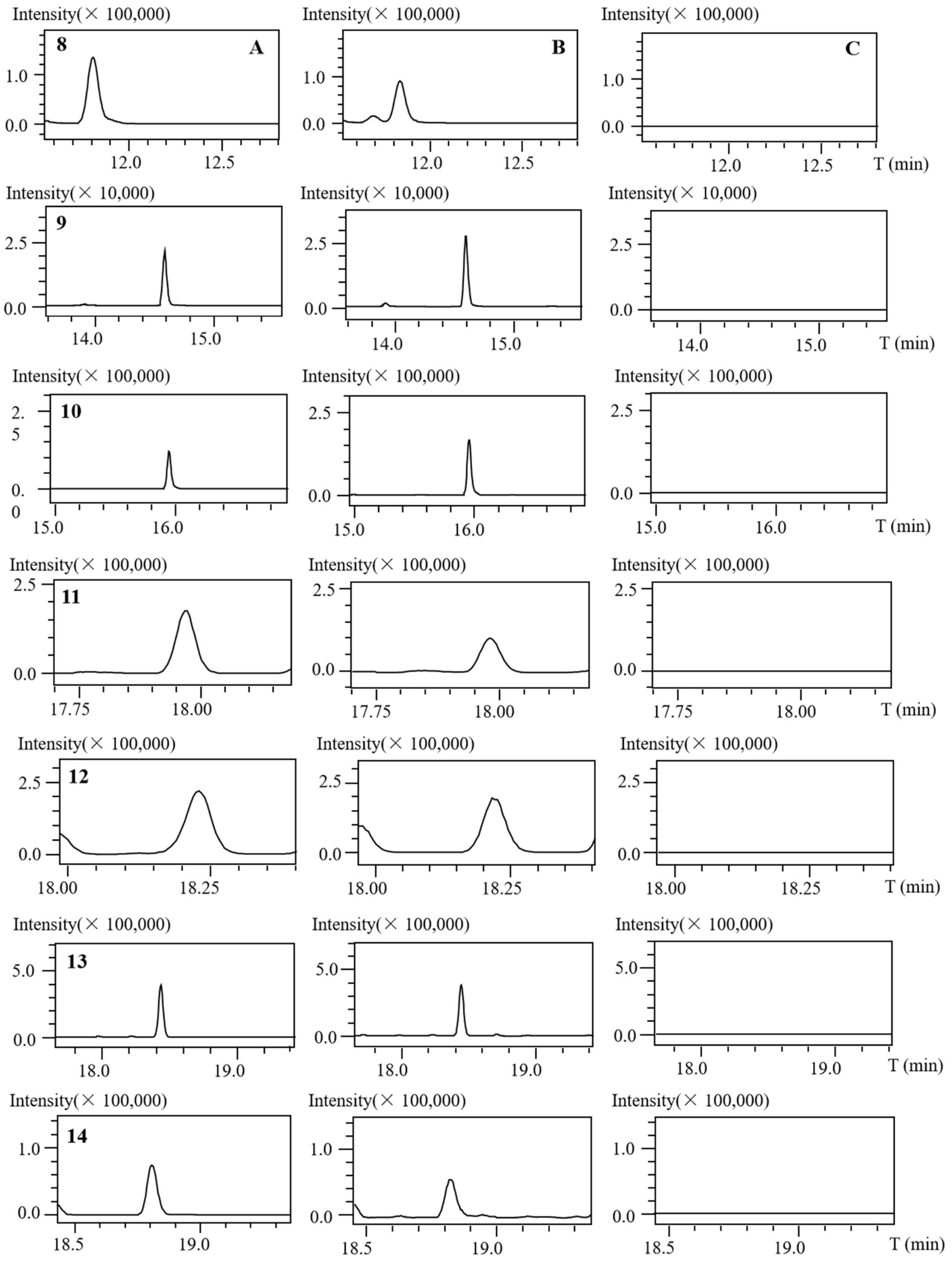

Figure S1 (continued). GC-MS SIM chromatogram of reference solution (A), sample solution (B) and blank control solution (C)

Note: 8: 4-Carene, 9: 2-Nonanone, 10: 4-Thujanol, 11: Borneol acetate, 12: Caryophyllene, 13: Terpinen-4-ol, 14: Myrtenal

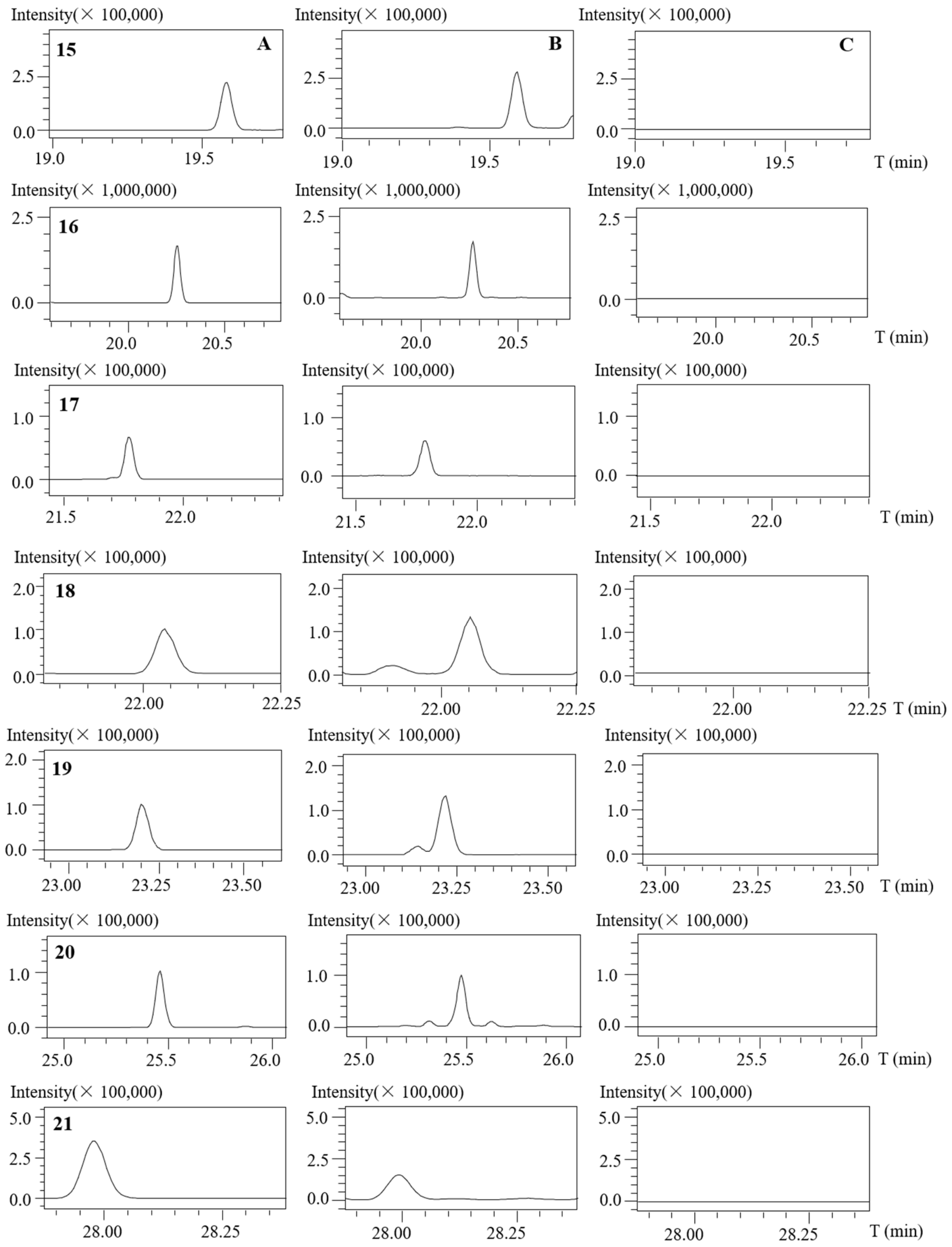

Figure S1 (continued). GC-MS SIM chromatogram of reference solution (A), sample solution (B) and blank control solution (C)

Note: 15: 4-(1-methylethyl)-2-Cyclohexen-1-one, 16: α-Terpineol, 17: Cuminaldehyde, 18: Myrtenol, 19: 2-(4-Methylphenyl)propan-2-ol, 20: Caryophyllene oxide, 21: Guaiol
